# Predictors of lost to follow up from antiretroviral therapy among adults in sub-Saharan Africa: a systematic review and meta-analysis

**DOI:** 10.1186/s40249-021-00822-7

**Published:** 2021-03-20

**Authors:** Hafte Kahsay Kebede, Lillian Mwanri, Paul Ward, Hailay Abrha Gesesew

**Affiliations:** 1 Clinical Pharmacy, College of Health Sciences, Defense University, Debrezeit, Ethiopia; 2 College of Medicine and Public Health, Flinders University, Adelaide, Australia; 3 Epidemiology Department, College of Health Sciences, Mekelle University, Mekelle, Ethiopia

**Keywords:** Antiretroviral therapy, Defaulting, Discontinuation, HIV, Lost to follow up, Meta-analysis, Systematic review, Sub-Sharan Africa

## Abstract

**Background:**

It is known that ‘drop out’ from human immunodeficiency virus (HIV) treatment, the so called lost-to-follow-up (LTFU) occurs to persons enrolled in HIV care services. However, in sub-Saharan Africa (SSA), the risk factors for the LTFU are not well understood.

**Methods:**

We performed a systematic review and meta-analysis of risk factors for LTFU among adults living with HIV in SSA. A systematic search of literature using identified keywords and index terms was conducted across five databases: MEDLINE, PubMed, CINAHL, Scopus, and Web of Science. We included quantitative studies published in English from 2002 to 2019. The Joanna Briggs Institute Meta-Analysis of Statistics Assessment and Review Instrument (JBI-MAStARI) was used for methodological validity assessment and data extraction. Mantel Haenszel method using Revman-5 software was used for meta-analysis. We demonstrated the meta-analytic measure of association using pooled odds ratio (*OR*), 95% confidence interval (*CI*) and heterogeneity using *I*^2^ tests.

**Results:**

Thirty studies met the search criteria and were included in the meta-analysis. Predictors of LTFU were: demographic factors including being: (i) a male (*OR* = 1.2, 95% *CI* 1.1–1.3, *I*^2^ = 59%), (ii) between 15 and 35 years old (*OR* = 1.3, 95% *CI* 1.1–1.3, *I*^2^ = 0%), (iii) unmarried (*OR* = 1.2, 95% *CI* 1.2–1.3, *I*^2^ = 21%), (iv) a rural dweller (*OR* = 2.01, 95% *CI* 1.5–2.7, *I*^2^ = 40%), (v) unemployed (*OR* = 1.2, 95% *CI* 1.04–1.4, *I*^2^ = 58%); (vi) diagnosed with behavioral factors including illegal drug use(*OR* = 13.5, 95% *CI* 7.2–25.5, *I*^2^ = 60%), alcohol drinking (*OR* = 2.9, 95% *CI* 1.9–4.4, I^2^ = 39%), and tobacco smoking (*OR* = 2.6, 95% *CI* 1.6–4.3, *I*^2^ = 74%); and clinical diagnosis of mental illness (*OR* = 3.4, 95% *CI* 2.2–5.2, *I*^2^ = 1%), bed ridden or ambulatory functional status (*OR* = 2.2, 95% *CI* 1.5–3.1, *I*^2^ = 74%), low CD4 count in the last visit (*OR* = 1.4, 95% *CI* 1.1–1.9, *I*^2^ = 75%), tuberculosis co-infection (*OR* = 1.2, 95% *CI* 1.02–1.4, *I*^2^ = 66%) and a history of opportunistic infections (*OR* = 2.5, 95% *CI* 1.7–2.8, *I*^2^ = 75%).

**Conclusions:**

The current review identifies demographic, behavioral and clinical factors to be determinants of LTFU. We recommend strengthening of HIV care services in SSA targeting the aforementioned group of patients.

*Trial registration *Protocol: the PROSPERO Registration Number is CRD42018114418

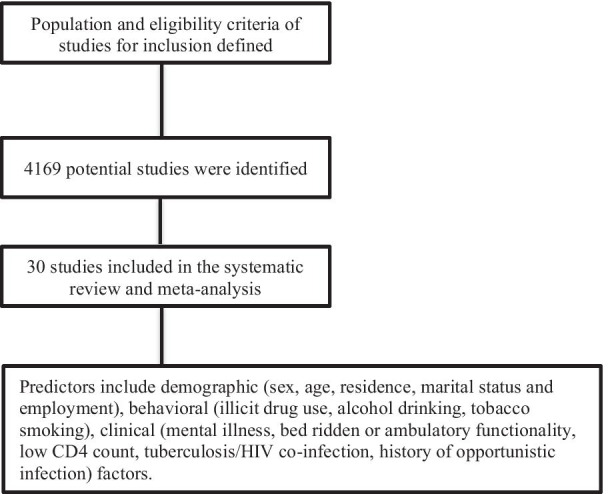

**Supplementary Information:**

The online version contains supplementary material available at 10.1186/s40249-021-00822-7.

## Background

Globally, the human immunodeficiency virus (HIV) has a significant proportion of population, with sub-Saharan Africa (SSA) contributing more than 2/3rd of the global infections (71%) [1]. By the end of 2018, HIV had caused more than 35 million deaths [1], destroyed global economy and more than 560 billion US dollars used in its prevention and mitigation [2]. By the year 2020, SSA was the hardest hit subcontinent recording 24.7 million infections. The advent of antiretroviral therapy (ART) has been a groundbreaking innovation to substantially reduce transmission and poor outcomes including poor quality of life, admission, and HIV related mortality [3].

In order to achieve positive HIV treatment outcomes, HIV infected individuals need to enroll in HIV care and to strictly adhere to prescribed ART regimes without interruption [4]. Nevertheless, multiple factors influence retention of patients, with lost to follow up from ART (hereon in referred to as LTFU) being one of the major problems [3, 5–7] in HIV care continuum. A variety of LTFU definitions exist. While some studies define LTFU as when the patient discontinue from ART for more than 3 months [3, 8–11] since the last visit to the clinic, others define LTFU as a discontinuation of 1 [12], 2 [5] or 12 [13] months. Evidence reveals that LTFU increases immunological failure, HIV-related complications, including AIDS-related re-admission, morbidity, mortality, and drug resistance [14–19]. The magnitude of LTFU in SSA ranges from 23.4% in South Africa [20] to 57.4% in Tanzania [21].

Several factors have been reported to influence LTFU in SSA including socio-demographic, behavioral, clinical and institutional factors [3, 5, 12, 20–24]. However, different studies have reported conflicting associations, and the meta-analytic association of these predictors is not well known. Despite the high HIV burden of disease in SSA, there are limited systematic reviews of risk factors associated with LTFU. For example, a systematic review conducted in 2015 revealed that the rate of retention in HIV care before ART eligibility was 23–88%. Patients including, women, individuals aged > 25 years, those with low CD4 count, individuals with high body mass index, individuals co-infected with tuberculosis, and those who had access to free cotrimoxazole treatment were more likely to remain in care [25]. Nevertheless, this study was conducted among the pre-ART population, and the authors did not report a meta-analytic association. Another systematic review and meta-analysis conducted in 2012 reported higher LTFU including in: men, patients with low CD4 cell counts, and patients with low socio-economic status [26]. However, this study was also conducted among the pre-ART population. Before the above reviews, a systematic review was conducted on patient retention in ART programs among the ART population in 2010 [27], but this study only reported the rate of LTFU and the risk factors were not assessed.

The current paper aims to synthesize the literature to improve the understanding of the risk factors for LTFU among adults in SSA. The study will provide evidence to inform effective policies and practices to enable the development of tailored and effective intervention strategies to the national HIV/AIDS control programs, and subsequently contribute to the success of the UNAIDs 95–95–95 treatment targets.

## Methods

### Study protocol

The present systematic review and meta-analysis was registered in a PROSPERO with PROSPERO Registration Number: CRD42018114418.

### Population

The review considered studies reporting on adult HIV-positive participants aged 15 years and older who had commenced ART—adult in ART care refers to age 15 years and above. We excluded transferred outpatients. Additionally, studies that did not provide data for adults and children separately were excluded from the analysis.

### Study design

A systematic review and meta-analysis were undertaken on studies conducted in the English language in SSA between 2002 and 2019. The year 2002 was selected as the time to commence the search strategy because of its significance, being the time period when ARTs were introduced in many SSA countries [28, 29].

### Outcomes

The review considered studies that included LTFU. Patients were considered “LTFU” when they were on ART treatment and missed at least one clinical appointment (1 month) but had not yet been classified as “dead” or “transferred out” [30].

### Exposures

The review considered studies that assessed risk factors for LTFU. These included age, sex, educational status, place of residence, matrimonial status, HIV status disclosure, partner's HIV status, HIV related stigma, smoking tobacco, drinking alcohol, tuberculosis HIV (TB/HIV) co-infection, isoniazid (INH) prophylaxis provision, cotrimoxazole or opportunist infection (OI) prophylaxis provision, presence of side effects, baseline CD4 counts, baseline WHO clinical stage, baseline functional status, regimen substitution, distance from the facility and facility type.

### Search strategy

An initial search was conducted across Google Scholar and MEDLINE followed by an analysis of the text words contained in the title and abstract, and of the index terms used to describe the article. A systematic search strategy which was followed by the initial analysis used all identified keywords and index terms across the five databases: MEDLINE, PubMed, CINAHL, Scopus, and Web of Science. Bibliographies of all articles were reviewed to identify additional relevant studies. Keywords for this review included “antiretroviral therapy”, “discontinuation”, “lost to follow up”, “retention”, “stopping medication”, “interruption” and all lists of sub-Saharan Africa countries. More detailed information is presented in Additional file 1: Table S1.

### Study selection and quality assessment

**Fig. 1:**
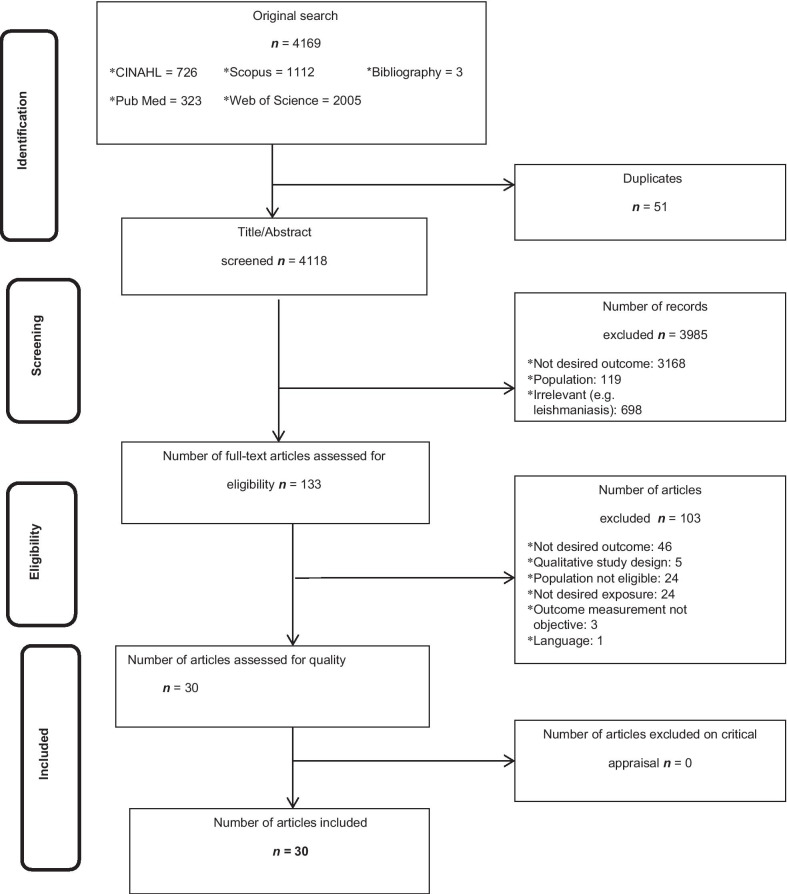
Preferred reporting items for systematic reviews and meta-analyses (PRISMA) flowchart detailing the identification and selection of studies for inclusion in the review. It describes the flowchart of search strategy

Only quantitative studies conducted in SSA and designed as cross-sectional studies, prospective and retrospective cohort studies, case–control studies, case series, clinical trials and individual case reports were included. Articles were retained if at least one search term for the outcome concept was found. Articles that did not meet all eligibility criteria were excluded and reasons were noted (Fig. 1). The retained papers were then assessed by two independent reviewers, HKK and HAG, for methodological rigour using standardized critical appraisal instruments from the Joanna Briggs Institute Meta-Analysis of Statistics Assessment and Review Instrument (JBI-MAStARI) (Additional file 1: S1 doc). The appraisal form comprised nine questions about the quality of the studies. Authors of primary studies were contacted to clarify missing or unclear data. Any disagreements between the reviewers were resolved through discussion or consulting a third reviewer.

### Data extraction

Data were extracted from papers included in the review using the standardized data extraction tool from JBI-MAStARI (Additional file 1: S2 doc). The extracted data included the following points: exposure, populations, study methods, and outcomes of significance to the review question and specific objectives.

### Data synthesis

The quantitative data were abstracted into an Excel spreadsheet and included details of study design, outcome and its measurement, sample size, number of participants with and without the event (LTFU) by the exposures of interest, and summary of the study. Clinical heterogeneity was assessed by the authorship team and was acceptable to add each outcome to meta-analysis. Statistical heterogeneity was assessed statistically using the standard Chi-square and *I*^2^ tests, with significant heterogeneity detected at the *P*-value < 0.05. Meta-analyses were conducted separately for LTFU and each exposure of interest using RevMan-5 Software (Version 5.3, The Nordic Cochrane Centre, Copenhagen) [31]. Meta-analysis was considered if *I*^2^ was below 85% [31]. Mantel Haenszel statistical method was used to calculate effect sizes, and forest plots to describe for the meta-analyses of exposures of interest with the event.

Pooled odds ratio (*OR*) [32] estimates and their 95% confidence intervals (*CI*) was calculated using random or fixed effect meta-analysis based on the degree of heterogeneity and number of studies [31, 33, 34]. Fixed effect meta-analysis was used if no heterogeneity was found and random effect if the heterogeneity was moderate (*I*^2^ < 85%) [31]. However, fixed effect model was considered irrespective of the degree of heterogeneity if the number of studies that reported LTFU was small (< five) [33, 34]. Pooling was considered when at least two studies assessed the outcome and the exposure of interest. Sensitivity analysis was considered and publication bias was assessed using funnel plot. This review was reported using PRISMA reporting guidelines for systematic review [35] (Additional file 1: S3 doc).

## Results

### Characteristics of studies

Four thousand one hundred and sixty-nine potential studies were identified. Fifty-one duplicated records and 3985 records were excluded after screening (Fig. 1). Full texts were obtained for 133 articles, of which 103 were further screened and excluded for various reasons as follows: 46 articles did not report on the desired outcome, five were qualitative studies designs, 24 studies did not have the eligible population, 24 studies did not report on the exposures of interest, three studies did not utilize objective measurement of outcome, and one study was not in English. Finally, 30 studies met the inclusion criteria and were assess to establish the association between LTFU and at least one of the exposures of interest. Out of the 30 studies included, two were from Central Africa, 17 from Eastern Africa, eight from Southern Africa, and three from Western Africa. The characteristics of these studies are described in Additional file 1: Table S2. Twenty three studies (77%) were retrospective cohort studies [3, 11, 12, 36–56], four were case–control studies [5, 24, 57, 58], two prospective cohort studies [47, 57] and one cross sectional study [59]. All studies had relatively high sample size with the total sample size of 336,069 study participants.

### Methodological quality

Four case–control studies [5, 24, 57, 58] met seven out of nine JBI critical appraisal criteria (Additional file 1: Table S3). The study sample sizes were representative of all respective adults (similarity in course of their condition/illness) populations living with HIV/AIDS. Outcome was measured reliably and assessed using objectives criteria. Confounding factors were identified and mitigation strategies stated. Comparisons were made among groups and appropriate statistical method of analyses employed were well described. However, because these studies were case–control, appraisal based on adequate follow-up and analyses of withdrawals were not applicable.

Twenty-five cohort studies (prospective and retrospective) [3, 10–13, 20, 36–46, 48, 50–55, 60] were included for the methodological quality assessment and met eight out of nine JBI critical appraisal criteria (Additional file 1: Table S3). The follow up of adults living with HIV/AIDS was carried out over a sufficient period. One cross sectional study [59] was included in the review and met seven out of nine JBI critical appraisal criteria. In all included studies, the study sample sizes were representative (similar in course of their condition/illness) of all adults living with HIV/AIDS. The outcome was measured reliably and assessed using objective criteria. Confounding factors were identified and strategies to deal with them were stated. Comparisons were made among groups and appropriate statistical analyses were used in the study. However, since included studies were cohort, and the appraisal tool has questions based on the case control study design, the appraisal about, "has bias been minimized about the selection of cases and controls" was interpreted as "has bias been minimized concerning a selection of exposed and of unexposed adults living with HIV/AIDS".

Also, a summary of the risk of bias of the included studies was assessed based on the Agency for Healthcare Research and Quality (AHRQ) criteria (Additional file 1: Table S4). The extent of risk bias was almost similar, and the studies had 'low risk' bias in the majority of areas. Due to the inapplicability of the design nature of the studies, they had 'unclear risk' judgment in a few criteria assessing the bias.

### Measurement of LTFU

The ART follow up status was recorded differently within studies, as either LTFU, discontinuation, defaulting or disengagement. LTFU ranged from 1 to 12 months absence from clinical appointments across the studies, and definitions across the studies differed:

- Eight studies [3, 37, 38, 41–43, 48, 53] defined LTFU when the patient had been absent from the ART clinic for 3 or more months and had not been reported as dead, transferred or a voluntary interruption.
- Three studies [10, 11, 39] defined LTFU when a patient had been absent from the ART clinic for more than 3 months.
- Five studies [5, 44, 50, 52, 54] defined LTFU when a patient had not been seen in ART clinic for at least 2 months.
- Three studies [20, 40, 55] defined LTFU when a patient had missed scheduled appointment within 6 months after ART initiation or when there was no documented information in the data base about the patient returning for the 6-month scheduled visit.
- Three studies [13, 51, 60] defined LTFU when a patient had failed to visit ART clinic for more than 6 months and had not been classified as dead or transferred out.
- One study [45] defined LTFU when a patient who had disengaged with HIV care within the first 4 months of ART initiation.
- One study [12, 56] defined LTFU when adult patients were 1 month late for their appointment to pick-up their antiretroviral drugs.
- Two studies [36, 58] defined LTFU when patient missed all the first 12 months follow up visits after ART initiation.
- Two studies [24, 57] reported defaulting and both defined it as individuals who had missed two or more clinical appointments.
- One study [59] measured discontinuation defined as a patient discontinuing all antiretroviral medications for at least 1 month.
- One study [46] assessed disengagement and was defined as a patient stopping accessing care in an ART clinic for > 6 months after his or her last visit date.

### Factors associated with LTFU among adults living HIV/AIDS

The factors affecting LTFU were grouped into the following themes: socio-demographic and economic, behavioral, facility-related and clinical factors.

### Socio-demographic and economic predictors

The following socio-demographic factors: age, sex, place of residence, marital status, educational status, religion, family size, employment status, HIV disclosure status, partners HIV status, and substance use were analyzed to assess their relationship with LTFU. Age of patients enrolled in HIV care was significantly associated with LFTU. Seven of the eight studies [11, 41, 50, 51, 55, 60, 61] assessing age reported that younger aged patients were more likely to LTFU than older aged patients. Twenty-eight studies assessed the relationship between sex and LTFU. Of these, six studies [36, 41, 42, 51–53] found a significant association between sex and LTFU where women were at a higher risk of LTFU than men. Four studies [11, 37, 44, 50] identified lower educational status or lack of formal education to be associated with LTFU. Two studies [50, 51] reported that being unmarried (*P* < 0.001) was a predictor for LTFU. Three studies [24, 37, 57] revealed that being rural dweller was a risk factor for LTFU. Braitsten and colleagues [52] stated travel time to the clinic for more than an hour was a risk factor for LTFU. Employment and income were the economic factors reported to have association with LTFU. Four studies, [24, 44, 48, 50] found that being unemployed or having no income was associated with LTFU. Mberi and colleagues [20] have also reported private business ownership as a risk factor for LTFU (AHR: 13.9, 95% *CI* 2.81–69.06, *P* = 0.001).

### Behavioral predictors

Behavioral factors including HIV status disclosure and substance use were reported to be influential to LTFU. Two studies [37, 53] identified the association between HIV status disclosure with LTFU and discussed that patients who did not disclosed their HIV status to be more likely to fall under the LTFU category. Two studies [48, 57] showed that using substance or drugs was a risk factor for LTFU. For example, Deribe and colleagues [57] identified taking illicit drugs (cocaine, cannabis and IV drugs) (a*OR* = 0.02, 95% *CI* 0.003–0.17) and excessive consumption of alcohol (a*OR* = 6, 95% *CI* 3.3–11.1) to be risk factors for LTFU. On the other hand, Kiguba and colleagues [59] identified the use of alternative medicines (a*OR* = 2.18, 95% *CI* 1.06–4.47) to be independent risk factor for LTFU.

### Health facility related predictors

The following health facility-related factors: level of health facility, having part-time staff, duration of treatment, and year of enrollment were reported to be influential to LTFU. Two studies [11, 41] reported that patients who had been served in health facilities with part-time staff had a lower potential of remaining on treatment. Three studies [36, 40, 41] reported that patients enrolled in earlier years of launching of HIV care services are more likely to drop out. Eguzo and colleagues [40] identified that early calendar year enrollees [(2008 vs 2010–2013) AHR = 3.1, 95% *CI* 1.16–8.17; *P* = 0.02)] and [(2009 vs 2010–2013) (HR = 2.69, 95% *CI* 1.05–6.9, *P* = 0.04)] were at high risk of LTFU than later year enrollees. One study [59] reported that patients on their earlier ART period (i.e. shorter duration of stay in ART) was highly associated with LTFU. HIV patients who had been on ART were eleven times to lost from care during their 1 year (*OR* = 11.1, 95% *CI* 5.00–25.00).

### Clinical predictors

Clinical factors including partner’s HIV status, WHO clinical stage, baseline CD_4_ counts, opportunistic infections, body mass index (BMI) level, baseline functional status, TB/HIV co-infection, mental status, presence of side effects, baseline hemoglobin level, INH prophylaxis provision, virologic failure, regimen substitution were reported to have a significant association with LTFU. Deribe and colleagues [57] also noted that partner’s sero-discordant (a*OR* = 3.5, 95% *CI* 1.1–11.1) or unknown HIV status (a*OR* = 1.7, 95% *CI* 1.02–2.9) were associated with LTFU. Study conducted by Asefa and colleagues [24] showed that patients whose partners: were discordant (a*OR* = 5.1; 95% *CI* 1.59–16.63), had unknown HIV status or not tested (a*OR* = 2.8; 95% *CI* 1.2–6.5) and had concerns with stigma (a*OR* = 8.3; 95% *CI* 2.9–23.8) were highly likely to exit from treatment.

Five studies [11, 20, 50, 52, 61] reported that patients who were known to have advanced WHO clinical stage (stage 3 or 4) were more likely to be LTFU. Mberi and colleagues [20] identified that patients with known advanced WHO treatment stage (AHR = 2.0, 95% *CI* 1.2–3.3, *P* = 0.006) were more likely to interrupt treatment. Other studies identified that being bedridden (a*OR* = 5.7, 95% *CI* 1.6–20.2) [57], ambulatory functional status (AHR = 1.9, 95% *CI* 1.2–3.06) [48] were predictors of LTFU. Study reported by Mekonnen and colleagues [48] revealed that having lower CD_4_ count in the last visit was associated with LTFU. Kaplan and colleagues [46] found that the probability of being lost from care increased as patient’s recent CD_4_ count decreased. Two studies [20, 50] discussed that virologic failure or having detectable viral load on ART was associated with LTFU. Similarly, Gesesew and colleagues [42] discussed that having an immunological failure on ART had greater than 2 times risk of LTFU (a*OR* = 2.3, 95% *CI* 1.9–8.2).

Two studies [42, 48] discussed that having an opportunistic infection was associated with LTFU. Gezae and colleagues [43] reported that any history of opportunistic infection(s)(OI/s) (AHR = 3.795; 95% *CI* 1.165–12.364) was a risk factor for LTFU. Gesesew and colleagues [42], revealed that Tuberculosis coinfection was associated with LTFU. Two studies [11, 38] identified that receiving isoniazid preventive therapy (AHR = 0.11; 95% *CI* 0.06–0.18) was associated with LTFU. One study by Asefa and colleagues [24] reported that patients with psychiatric illness had around five times (a*OR* = 4.7; 95% *CI *1.65–13.35) probability of LTFU.

Two studies [11, 48] discussed BMI to be associated with LTFU but found conflicting results. While Mekonnen and colleagues [48] reported being underweight was associated with LTFU, Teshome and colleagues [11] pointed out that being within the normal body weight range (AHR = 0.6, 95% *CI* 0.4–0.9) was associated with lost from treatment. Study reported by Kaplan and colleagues [46] identified that LTFU was associated with ART regimen with the use of stavudine at last visit (AHR = 1.72; 95% *CI* 1.57–1.89) being one of particular risk factors for termination from the HIV care. Mberi and colleagues [20] identified that patients with a history of ART adverse event had a lower risk (AHR = 0.6, 95% *CI* 0.4–0.9, *P* = 0.044) of becoming LTFU than those that had not.

### Meta-analysis of factors affecting LTFU from ART care

**Fig. 2:**
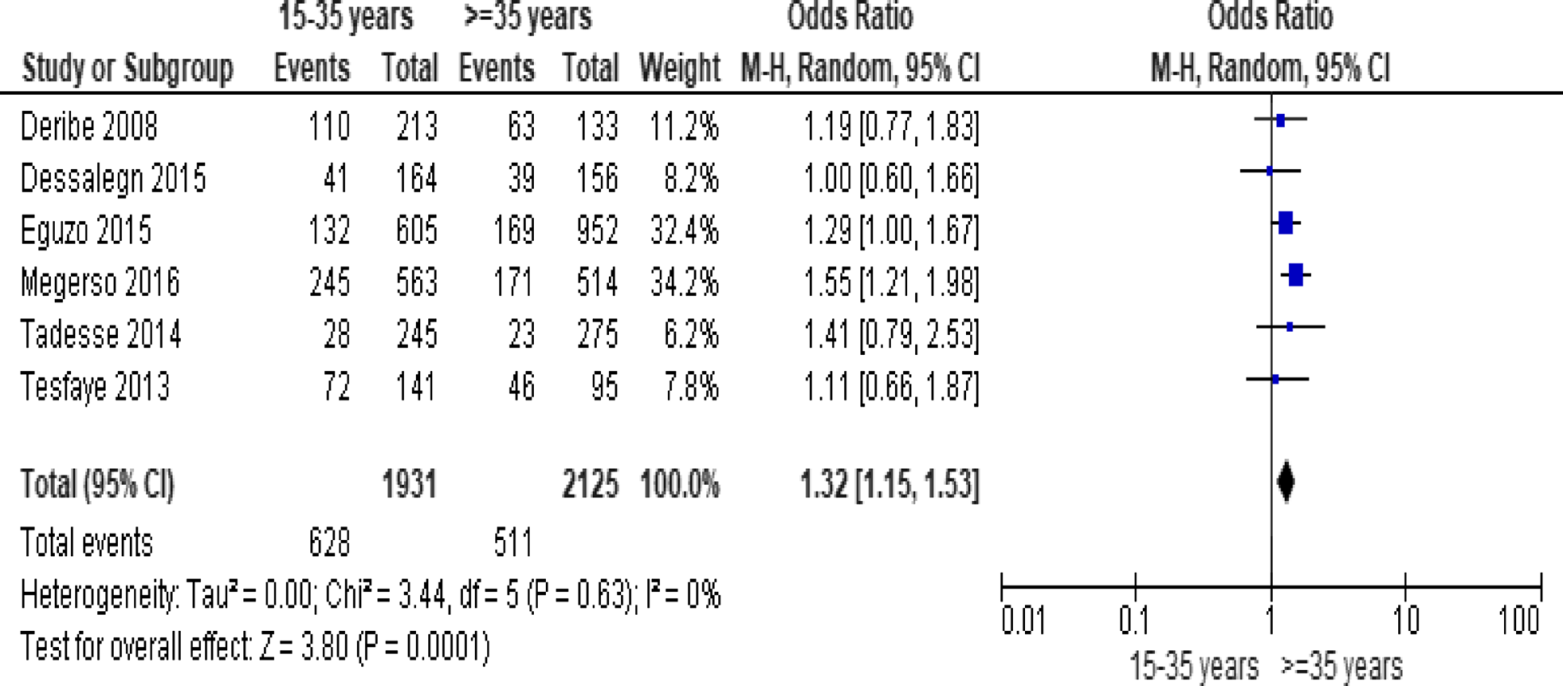
Forest plot of the meta-analytic association between age and LTFU. It shows that the risk of loss to follow up is higher in younger patients relative to their older counterparts

**Fig. 3:**
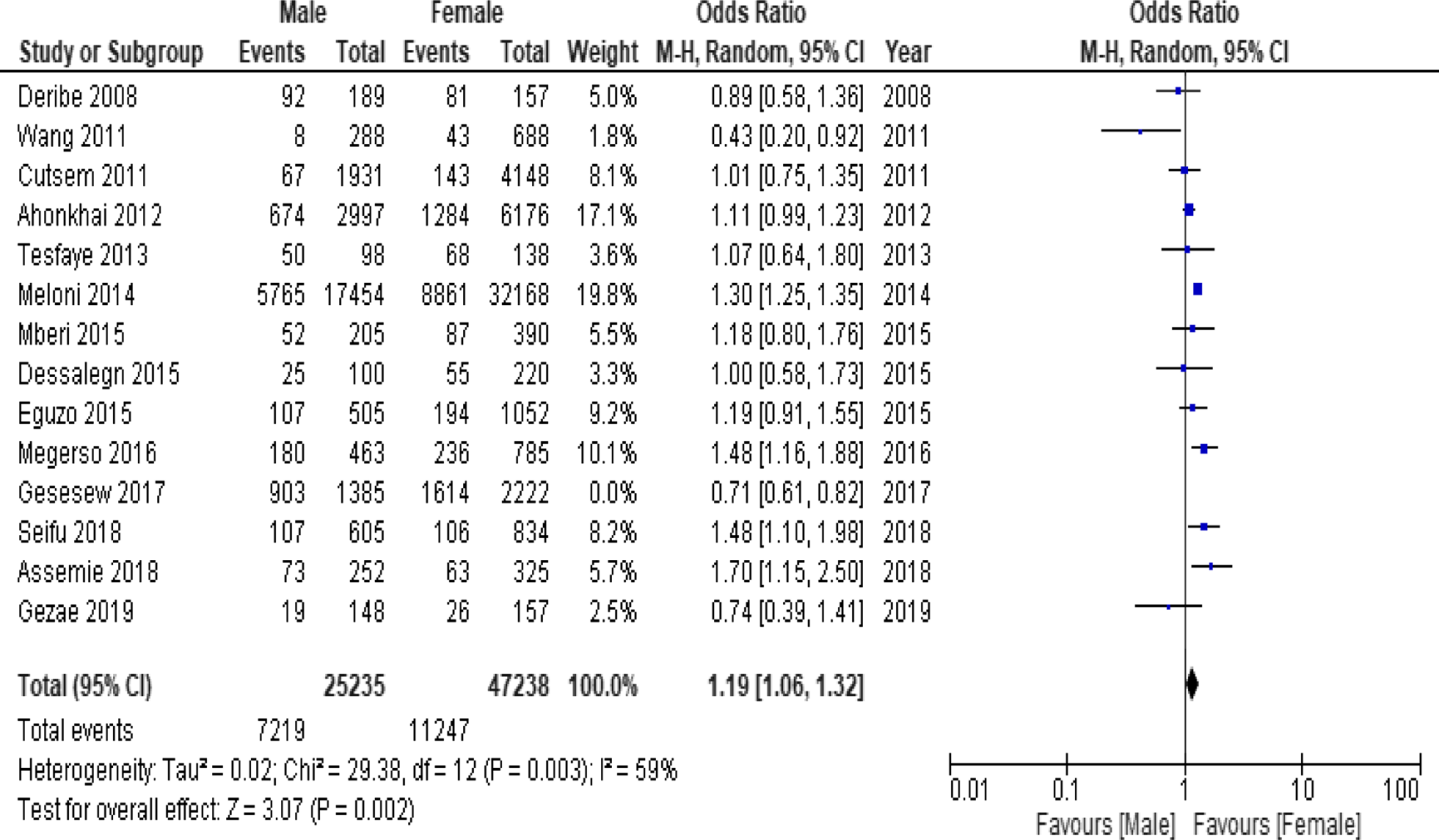
Forest plot of meta-analytic association between sex and LTFU. It shows that the risk of lost to follow up is higher among males than female patients

**Fig. 4:**
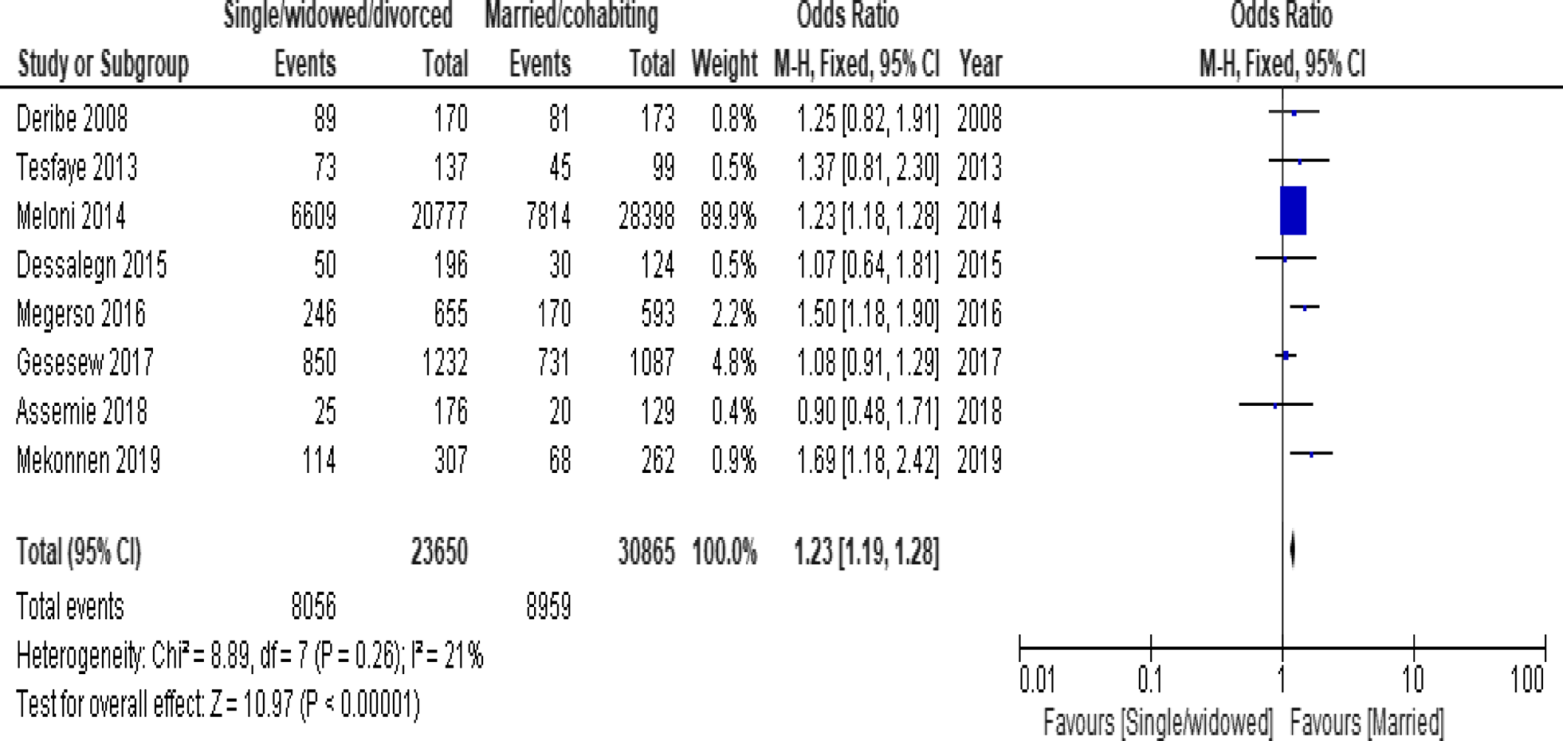
Forest plot of meta-analytic association between marital status and LTFU. It shows that the risk of lost to follow up is higher among single or widowed or divorced than married

**Fig. 5:**
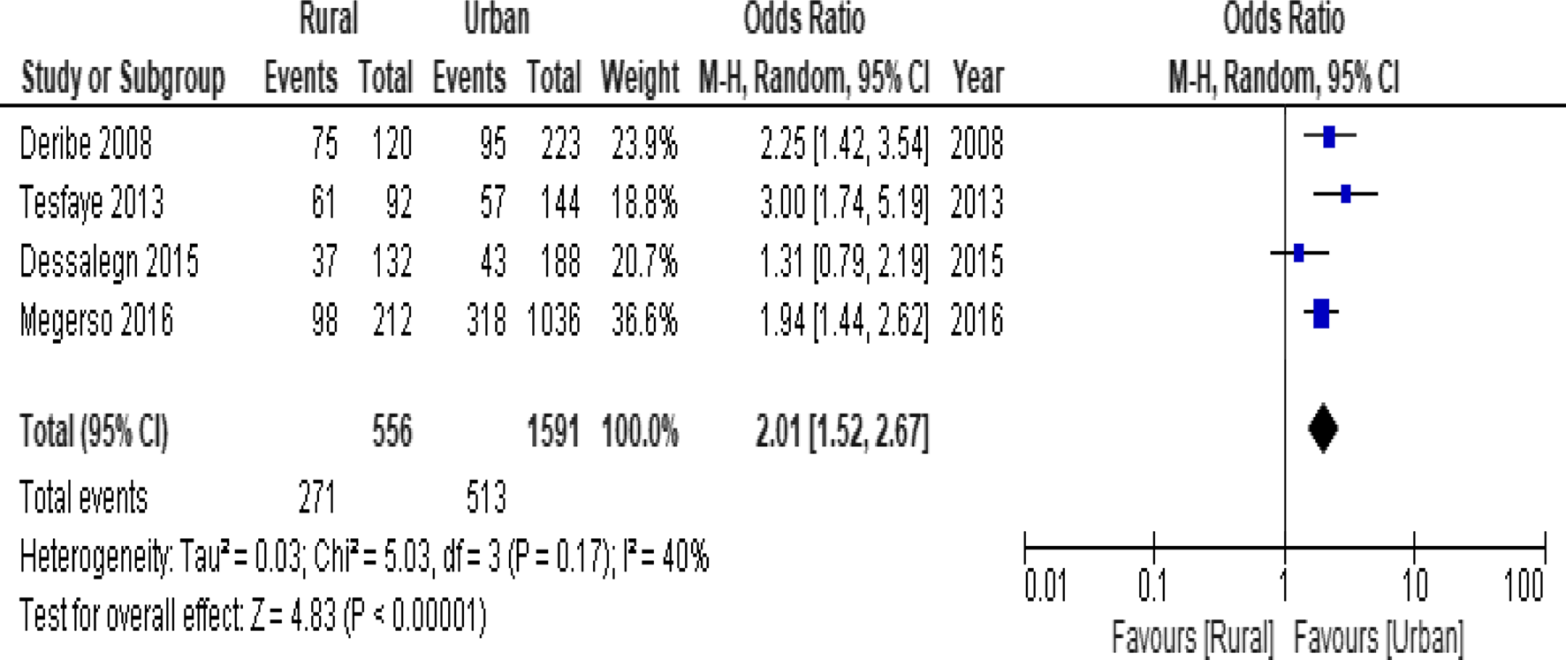
Forest plot of meta-analytic association between residence and LTFU. It shows that the risk of lost to follow up is higher in rural than urban residents

**Fig. 6:**
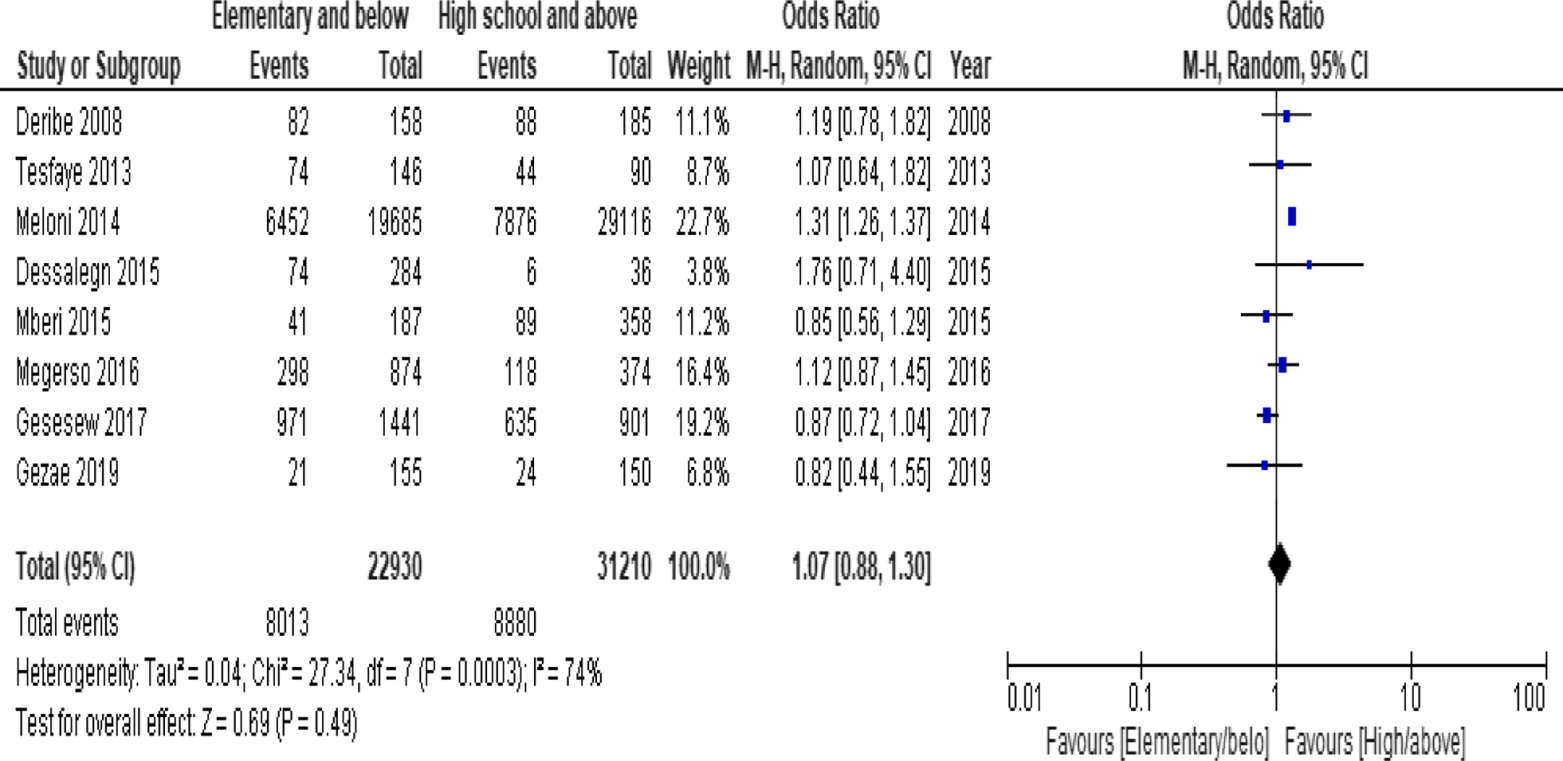
Forest plot of meta-analytic association between educational status and LTFU. It shows that the risk of lost to follow up is not associated with literacy level

**Fig. 7:**
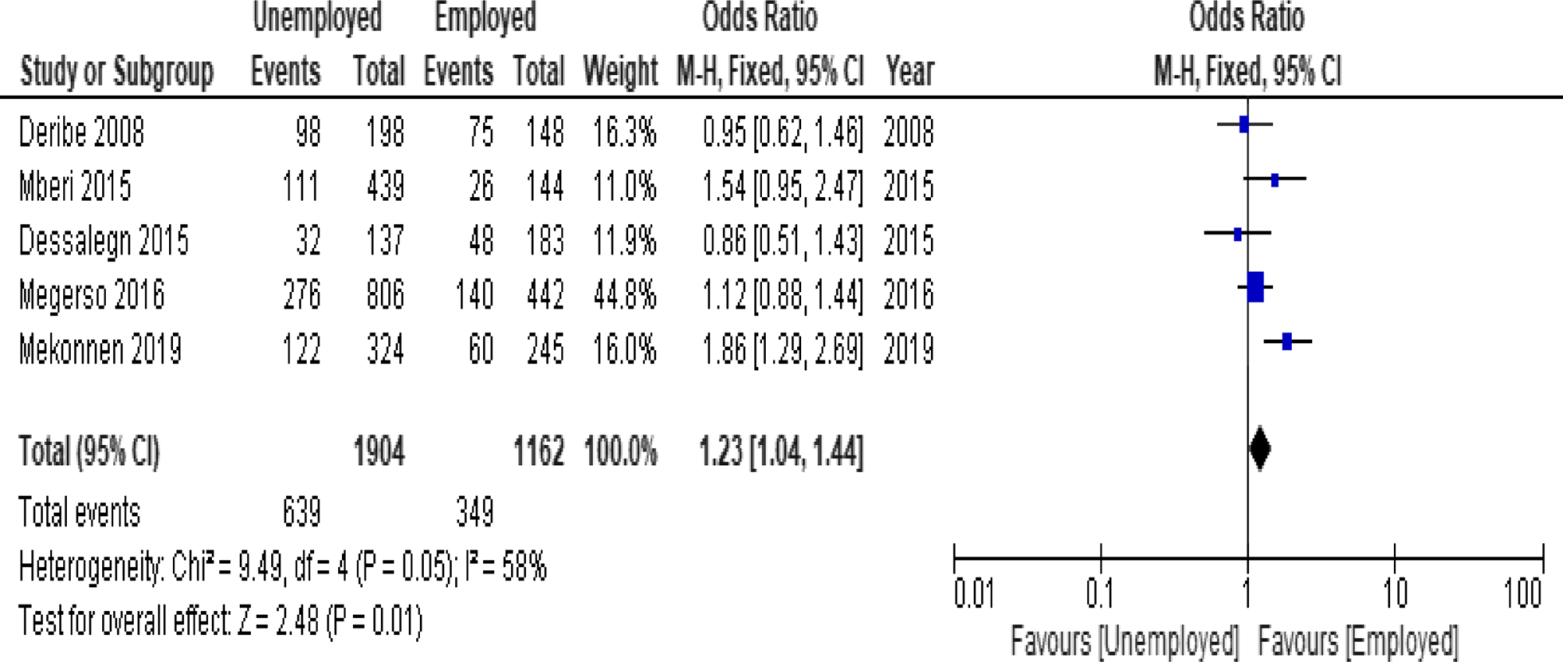
Forest plot of meta-analytic association between employment and LTFU. It shows that the risk of lost to follow up is higher in unemployed patients

**Fig. 8:**
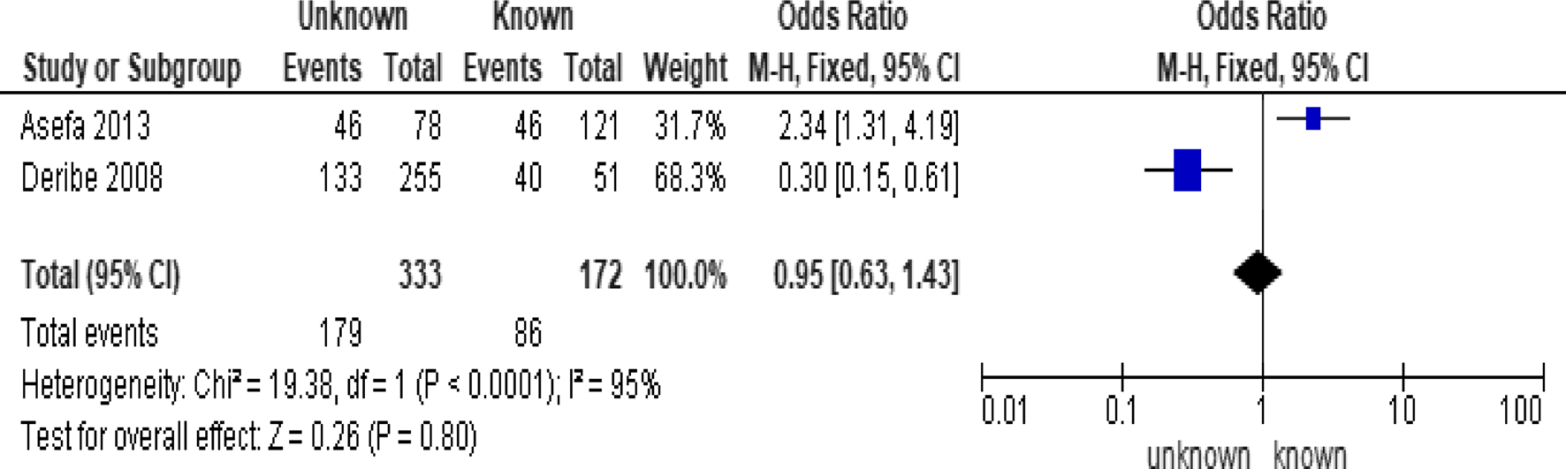
Forest plot of meta-analytic association between partners HIV status and LTFU. It shows that the studies show high heterogenicity

When the meta-analysis was performed, several the socio-demographic and economic variables were found to have significant association with LTFU. Variables including younger age (Fig. 2; *OR* = 1.32, 95% *CI* 1.15–1.53, *n* = 6, *I*^2^ = 0%) and male patients (Fig. 3; *OR* = 1.19, 95% *CI* 1.06–1.32, *n* = 14, *I*^2^ = 59%) had higher odds of LTFU than their counter parts. Patients who were single or widowed or divorced (Fig. 4; *OR* = 1.23, 95% *CI* 1.19–1.28, *n* = 8, *I*^2^ = 21%) and those who were from rural dwellings (Fig. 5; *OR* = 2.01, 95% *CI* 1.52–2.67, *n* = 4, *I*^2^ = 40%) were more likely to be with the LTFU category, but no difference was found by educational status (Fig. 6). The odds of LTFU was higher in unemployed patients (Fig. 7: *OR* = 1.23, 95% *CI* 1.04–1.44, *n* = 5, *I*^2^ = 59%) than their comparator. The odds of LTFU was not different by partner's HIV status (Fig. 8).

**Fig. 9:**
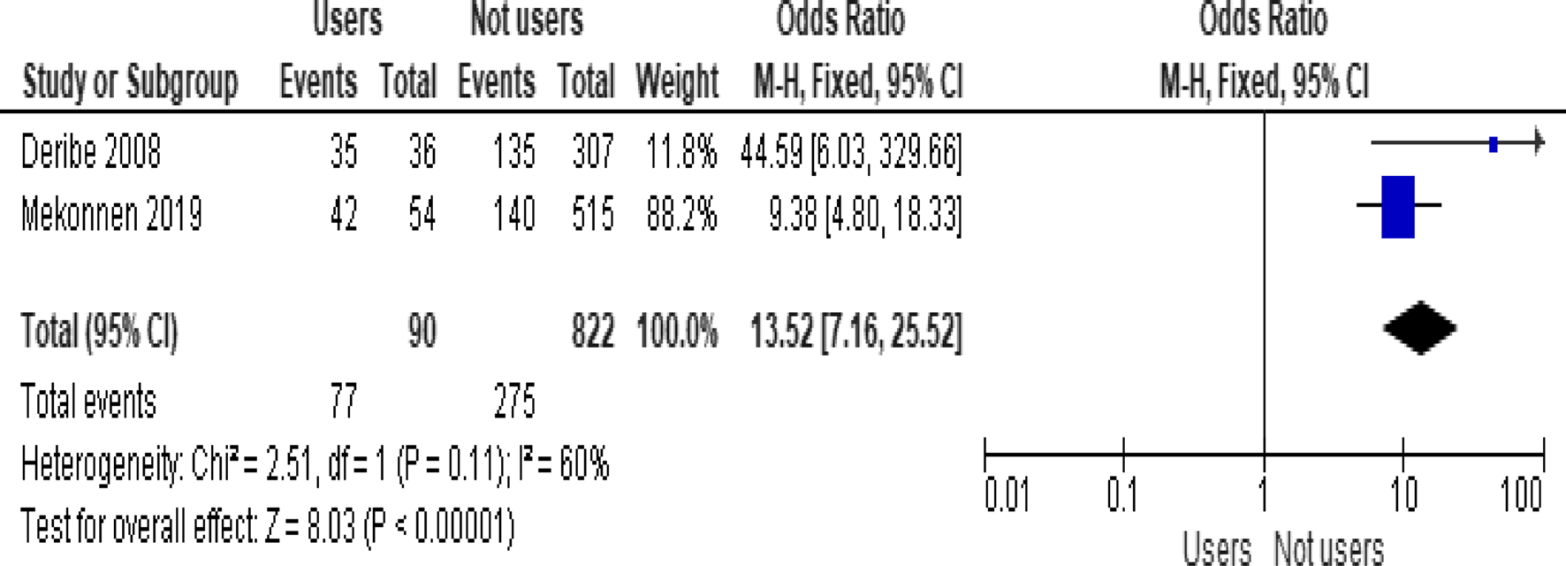
Forest plot of meta-analytic association between hard drugs use and LTFU. It shows that the risk of lost to follow up is higher in patients who are hard drug uses

**Fig. 10:**
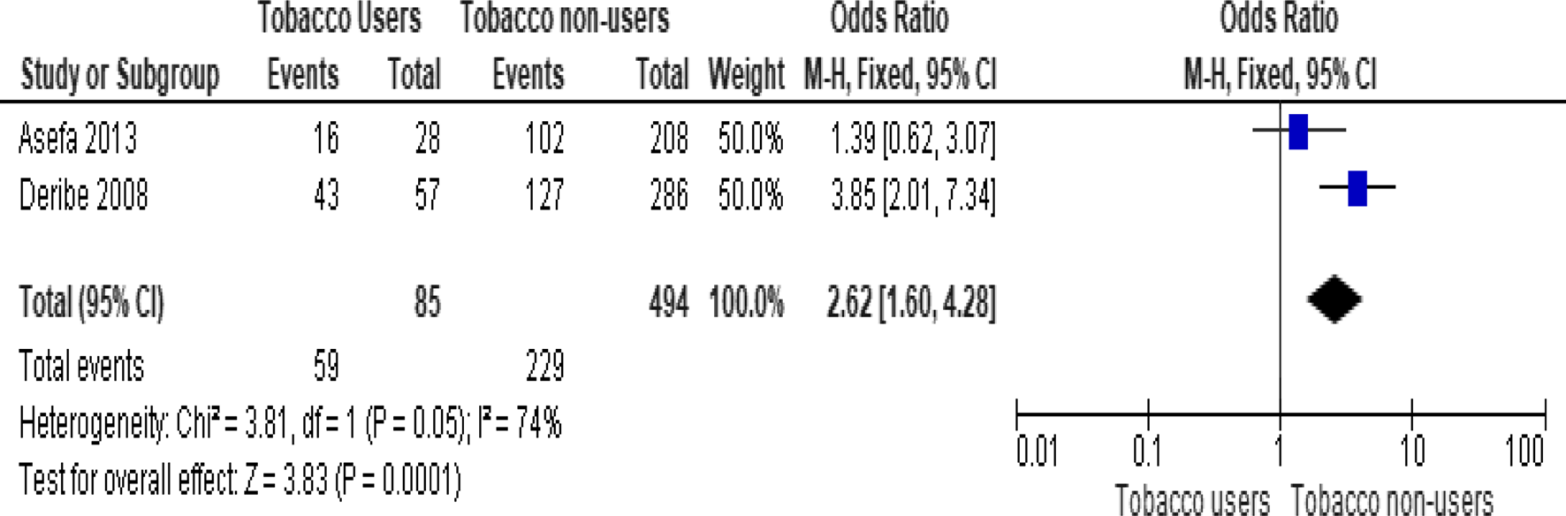
Forest plot of meta-analytic association between tobacco smoking and LTFU. It shows that the risk of lost to follow up is higher in patient’s tobacco smokers

**Fig. 11:**
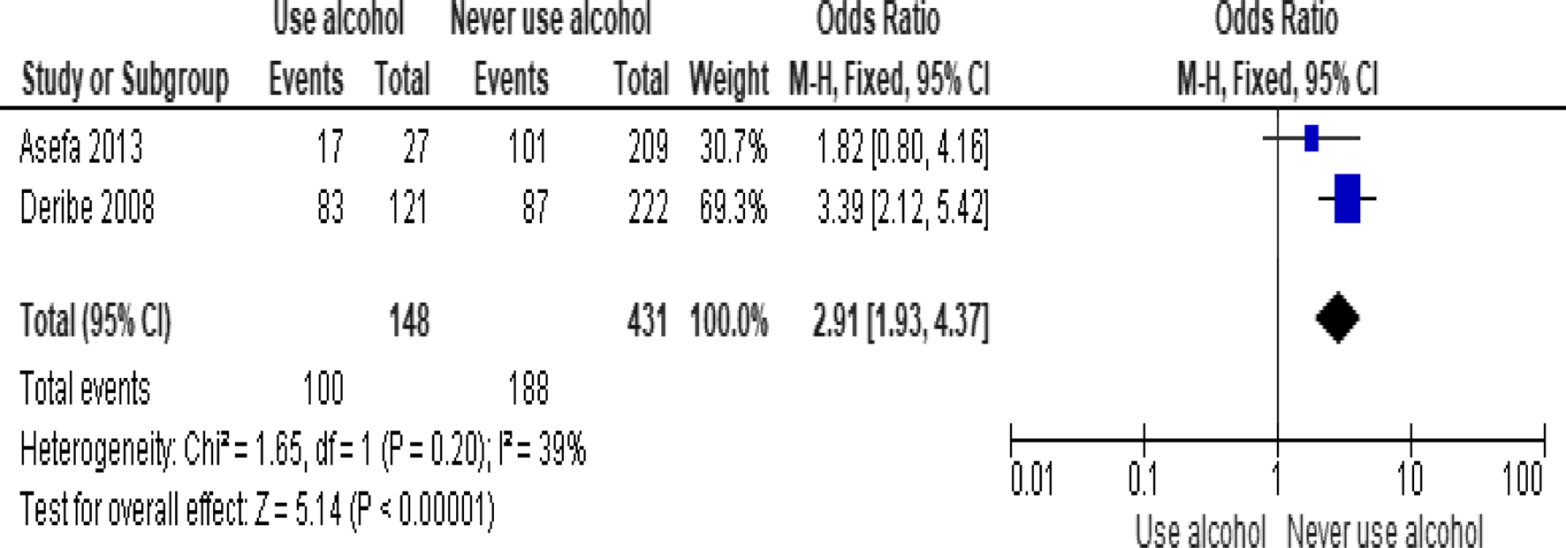
Forest plot of meta-analytic association between alcohol drinking and LTFU. It shows that the risk of lost to follow up from ART is higher in patients who drink alcohol

Of the behavioral factors, the meta-analysis association suggested a significant association between hard drugs use and LTFU (Fig. 9; *OR* = 13.52, 95% *CI* 7.16–25.52, *n* = 2, *I*^2^ = 60%). Tobacco smoking (Fig. 10; *OR* = 2.62, 95% *CI* 1.60–4.28, *n* = 2, *I*^2^ = 74%) and alcohol drinking (Fig. 11; *OR* = 2.91, 95% *CI* 1.93–4.37, *n* = 2, *I*^2^ = 39%) were also strongly associated with LTFU.

**Fig. 12:**
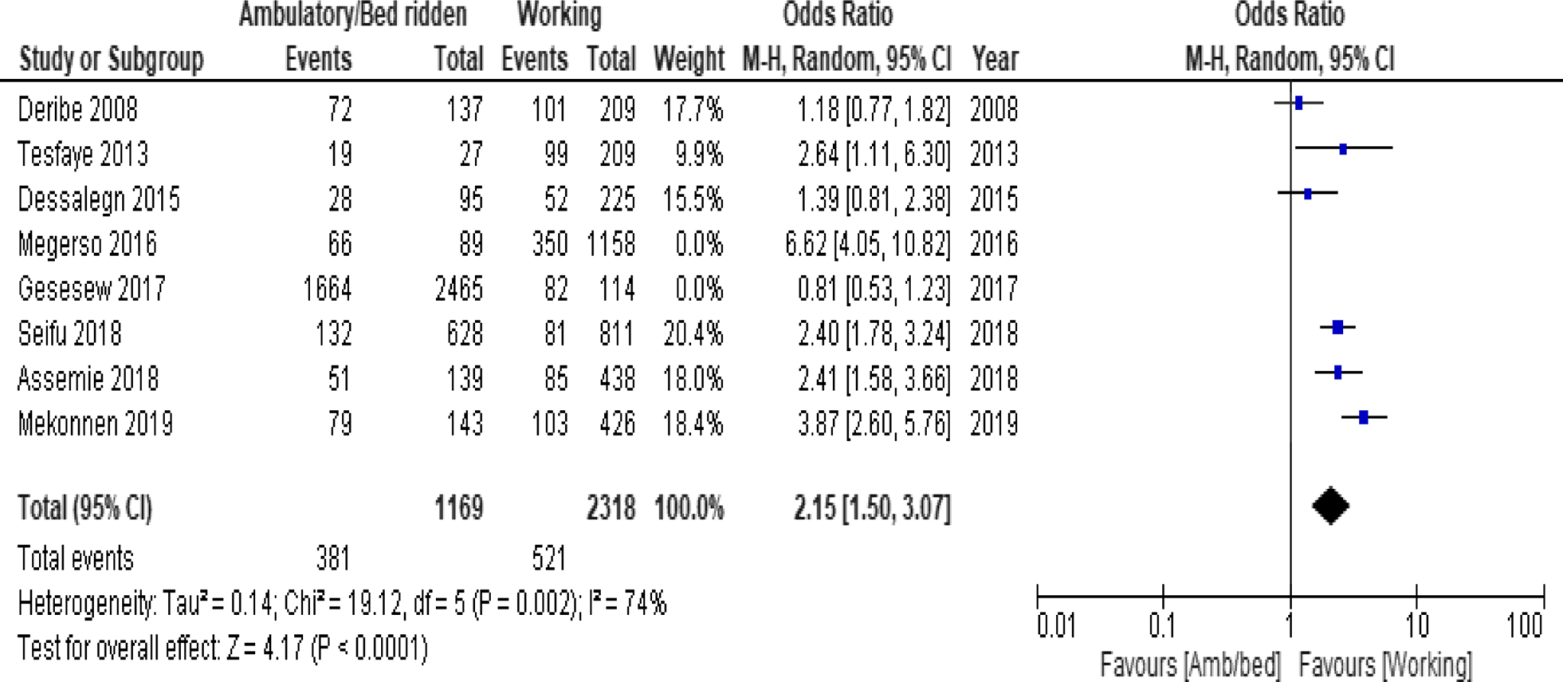
Forest plot of meta-analytic association between base line functional status and LTFU. It shows that the risk of lost to follow up is higher in bed ridden and ambulatory than working patients

**Fig. 13:**
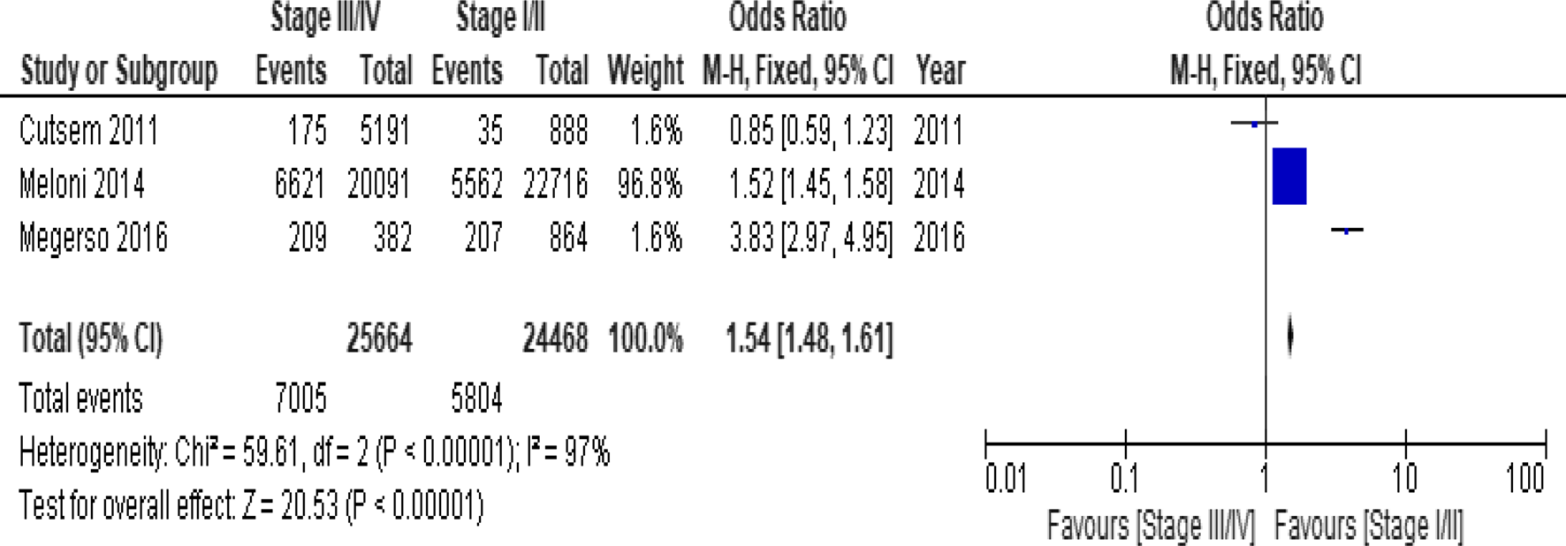
Forest plot of meta-analytic association between WHO clinical stage and LTFU. It shows that the studies show high heterogenicity

**Fig. 14:**
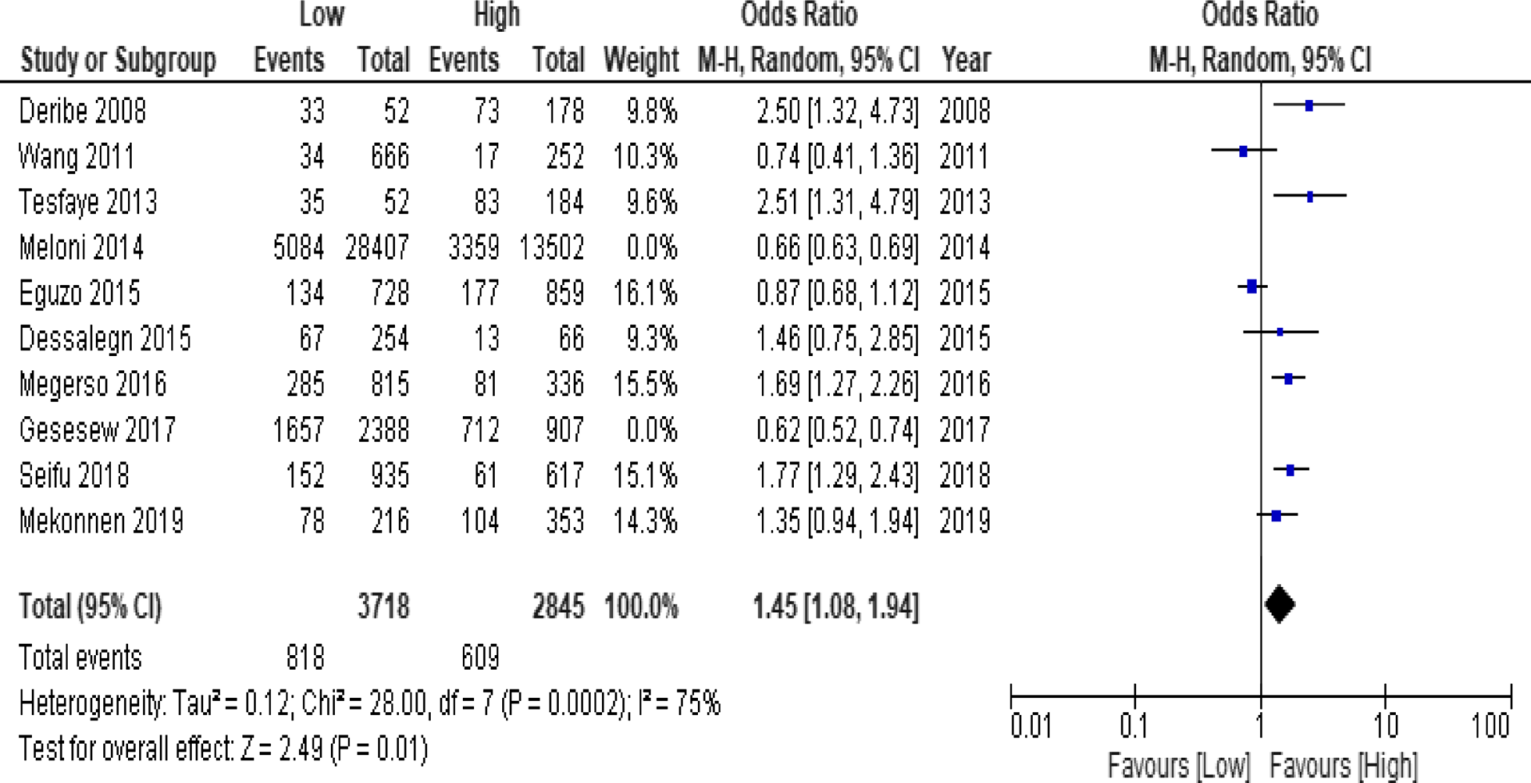
Forest plot of meta-analytic association between base line CD4 count and LTFU. It shows that the risk of lost to follow up is higher in patients with low than high CD4 count

**Fig. 15:**
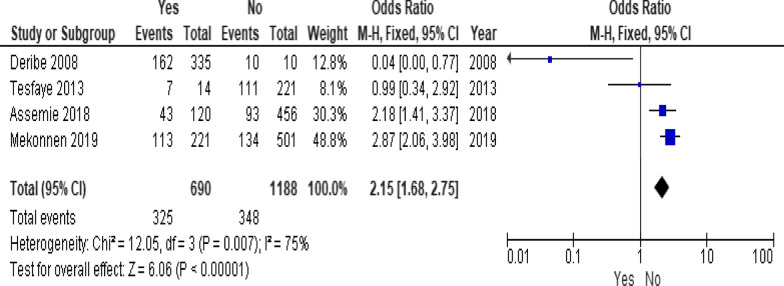
Forest plot of meta-analytic association between having history of OI treatment and LTFU. It shows that the risk of lost to follow up is higher in patients with history of OI treatment

**Fig. 16:**
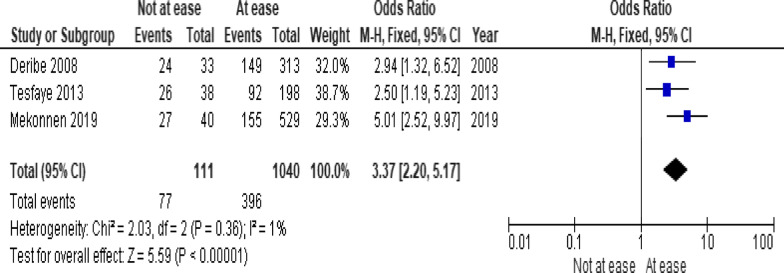
Forest plot of meta-analytic association between mental status and LTFU. It shows that the risk of lost to follow up is higher in patients with mental illness than their counterparts

**Fig. 17:**
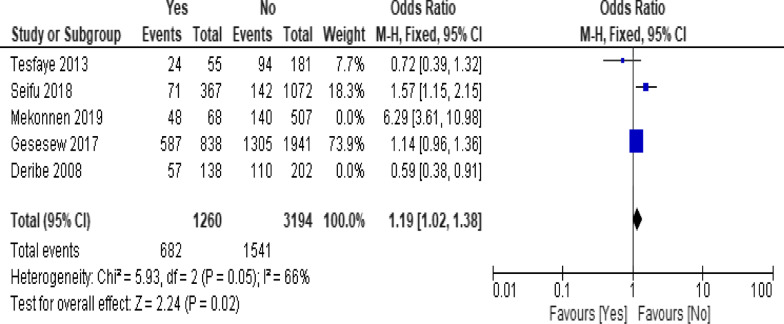
Forest plot of meta-analytic association between TB coinfection and LTFU. It shows that the risk of lost to follow up is higher in patients co-infected with TB

**Fig. 18:**
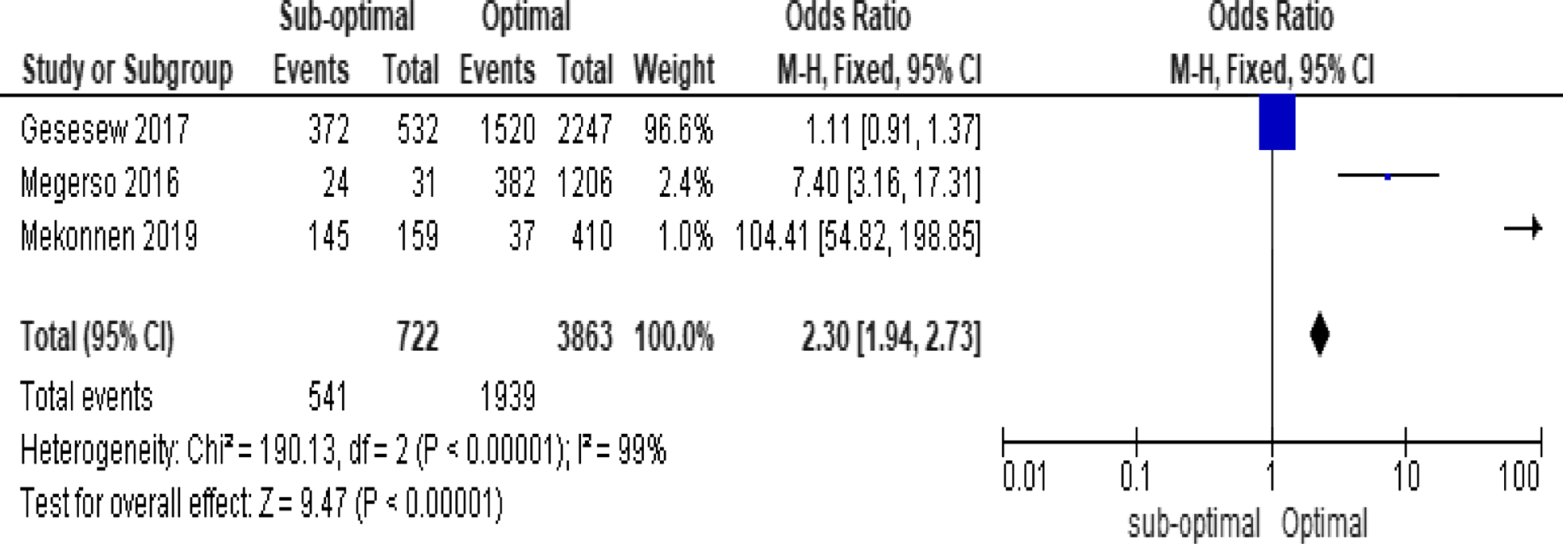
Forest plot of meta-analytic association between adherence status and LTFU. It shows that the studies show high heterogenicity

**Fig. 19:**
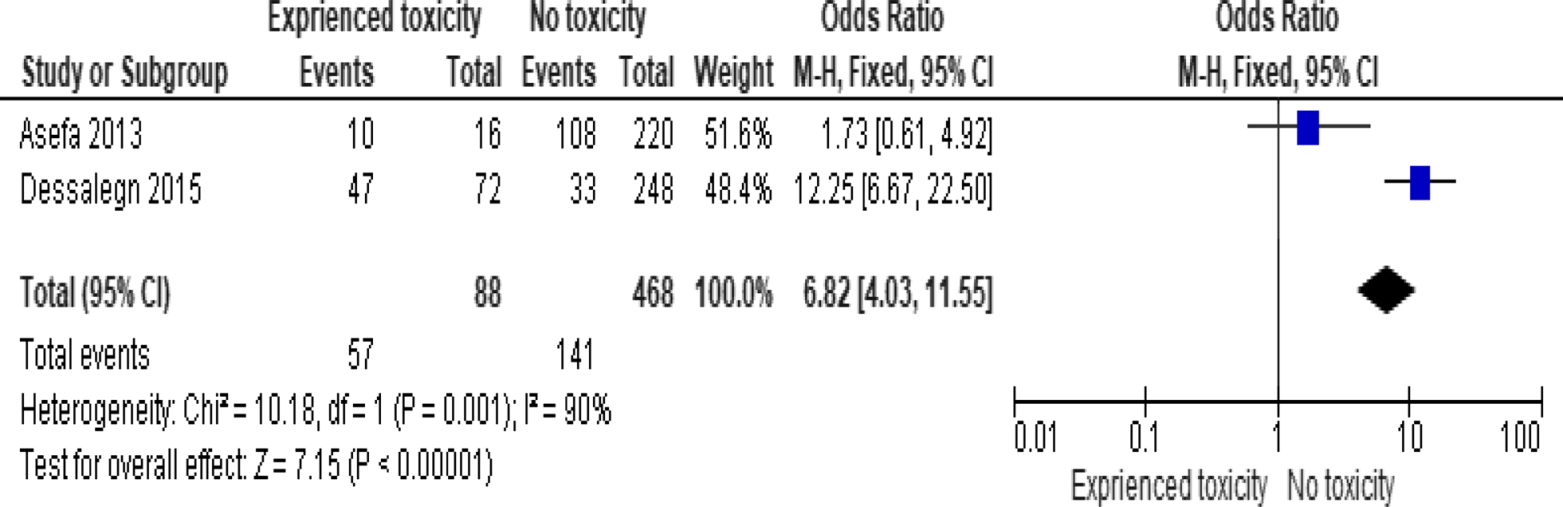
Forest plot of meta-analytic association between drug toxicity experience and LTFU. It shows that the studies have high heterogenicity

**Fig. 20:**
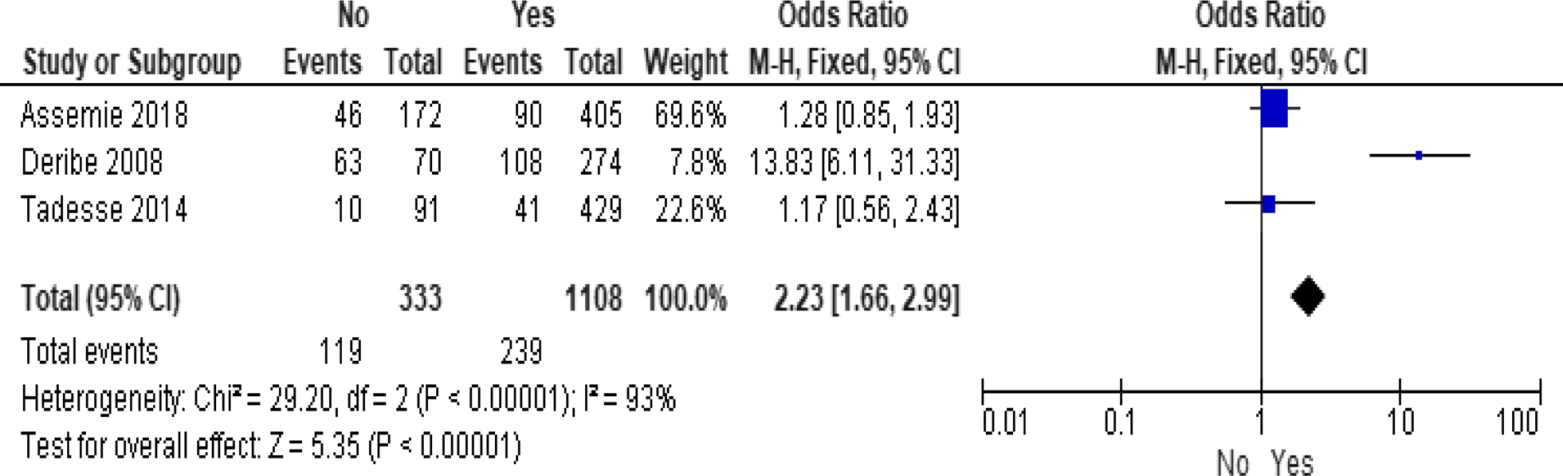
Forest plot of meta-analytic association between cotrimoxazole prophylaxis and LTFU. It shows that the studies have high heterogenicity

Of the clinical variables included in the meta-analysis, having ambulatory or bedridden functional status at entry had high probability of LTFU than having working functional status (Fig. 12; *OR* = 2.15, 95% *CI* 1.50–3.07, *n* = 8, *I*^2^ = 74%). As shown in Fig. 12, studies with 0% weights were removed from the meta-analysis calculation to prevent the introduction of significant heterogeneity. Advanced WHO clinical Staging was also found a predictor of LTFU although the studies were found heterogenious (Fig. 13; *OR* = 1.54, 95% *CI* (1.48–1.61), *n* = 3, *I*^*2*^ = 97%). Having low CD4 count in the last visit was also another significant barrier for LTFU (Fig. 14; *OR* = 1.45, 95% *CI* 1.08–1.94, *n* = 10, *I*^2^ = 75%). Patients with history of opportunistic infections treatment had higher risk of LTFU from care than those without (Fig. 15; *OR* = 2.15(1.68–2.75), *n* = 14, *I*^2^ = 75%). Patients who had mental illness (Fig. 16; *OR* = 3.37 (2.20–5.17), *n* = 3, *I*^2^ = 1%) had higher odds of LTFU from ART program compared to their counter parts. Active tuberculosis (TB) co-infection was associated with LTFU (Fig. 17; *OR* = 1.19, 95% *CI* 1.02–1.38, *n* = 5, *I*^2^ = 66%). As shown in Fig. 17, the article by Mekonnen and colleagues [48] and Deribe [57] were removed from the meta-analysis calculation to prevent the introduction of significant heterogeneity. Although the meta-analytic association between LTFU and adherence (Fig. 18), drug toxicity (Fig. 19) and cotrimoxazole prophylaxis (Fig. 20) is significant, substantial heterogenicity was found among the studies.

## Discussion

Studies in the current systematic review and meta-analysis identified many determinants of LTFU from ART programs among adult HIV positive patients in SSA. This systematic review and meta-analysis identified studies conducted in all zones of Africa. The study revealed that younger HIV positive patients were more likely to experience LTFU. In conformity with previous studies, this could probably be due to younger patients being eager to experiment substance usage, behaviours that are associated with being a youth [62–64]. Similar to previous studies, being a female was found to be a risk factor for LTFU [62], at a higher risk of stigma, and more likely to visit traditional healers than men [65, 66].

The residential location had a significant association with LTFU from the ART program, with rural dwellers being more likely to LFTU from care compared to their urban counterparts. It is reasonable to hypothesis that residing in rural areas could be a risk factor for LTFU because of poor access to health care including difficulties associated with transportation to services [67, 68] which are often concentrated in urban settings. This study shows that unemployed patients had increased risk of being LTFU compared with employed patients. It is reasonable to argue that unemployment would contribute to financial constraints, leading to inability of HIV patients to support essentials such as transportation, food, and other expenses [69]. Moreover, the existing myth in sub Saharan African communities that taking medication without proper food would be ineffective or even harmful [70] could also contribute to LTFU when HIV patients did not find continuing care meaningful while hunger and other challenges took a priority in their life.

The findings from meta-analysis suggested alcohol drinkers, illicit drug users and tobacco smokers to be at a high risk of LTFU from care. Consistent with other studies, this study supports evidence that substance usage causes poor compliance to taking ART drugs [71]. Moreover, it is also known that patients in HIV care often are marginalized and do encounter stigma and discrimination, factors that contribute to poor engagement with HIV care services [72–75].

Correspondingly, HIV patients who had poor outcomes i.e. advanced WHO clinical stage, bedridden and ambulatory functional status, virologic or immunologic failure or clinical failure at entry were at an increased risk of being LTFU. While it is hypothesised that poor health status of patients in this study would lead to a higher risk of developing opportunistic infections and thus poor attendance to HIV care services, previous studies have shown similar association [3, 69, 76]. Furthermore, tuberculosis co-infection was another important risk factor for LTFU in adult HIV patients. It is reasonable to hypothesize that patients with TB/HIV co-infection could have a higher burden of taking pill, adverse drug toxicities, and interactions among anti-TB and ART, complexities that would demand a higher commitment to following all the medications. Regarding the role that co-morbidities played in LTFU, the meta-analysis showed mental illness as a comorbidity was a strong predictor of LTFU from ART program. Evidently, HIV escalates lifetime prevalence of mental illness [77], and mental illness can interfere with ART adherence and retention [77, 78]. In addition, stigma and discriminating combined with mental health issues have been reported to deter patients from seeking HIV care [77].

While the current systematic review has provided significant insight of determinants of LTFU from ART care service, it is important to acknowledge that there could be several important gaps. Firstly, of the studies reviewed, there was no “gold standard” measurement method in the definition of LTFU [79]. Secondly, the study findings and interpretations should consider the following important limitations. All, except two of the included studies were retrospective studies. This implies that meta-analytic findings would be viewed as demonstrating an association, but not causally related. The search strategy was limited to English language which may have led to a reporting bias [80]. Transferred out cases were excluded and there were no records to indicate their attendance to care thereafter. However, we acknowledge that patients who were transferred out could have continued with HIV care elsewhere.

Efforts to contact authors of the corresponding studies were not successful and hence, we have been unable to report findings of meta-analytic association of the following variables: regimen substitution, regimen type, hemoglobin level, adherence concern, INH prophylaxis, year of ART initiation and facility type. We recommend similar study to be carried out among children with HIV, and HIV patients in second line ART regimens.

## Conclusions

This systematic has review provided high quality data on the predictors of LTFU among PLHIV adults in SSA. Several factors including drinking alcohol, smoking tobacco, having mental illness, being bed ridden or ambulatory functional status, low CD4 count, and TB co-infection predicted LTFU. The review makes a substantial contribution in providing information that will inform important strategies to retaining patients in HIV management programs especially in sub Saharan African national HIV/AIDS control programs.

## Supplementary Information

**Additional file 1:** **Table S1.** Full searching strategy by databases. **S1 doc.** JBI Critical Appraisal instruments. **S2 doc. **JBI Data extraction instruments. **S3 doc.** PRISMA Checklist of items to include when reporting a systematic review or meta-analysis. **Table S2.** Characteristics of included articles (n = 30). **Table S3. **Assessment of methodological quality (n = 30). **Table S4.** Risk of Bias Assessment within the studies (n = 30)

## Data Availability

We browse the reference list of included articles and raw data from publicly available databases and websites. The statistical analysis software material (RevMan-5 Software) is publicly available from the Cochrane library [81].

## Abbreviations

AIDS: Acquired immune deficiency syndrome
AHR: Adjusted hazard ratio
AHRQ: Agency for health care quality and research
A*OR*: Adjusted odds ratio
ART: Antiretroviral therapy
*CI*: Confidence interval
HAART: Highly active anti-retroviral therapy
HIV: Human immunodeficiency virus
JBI-MAStARI: Joanna Briggs Institute Meta-Analysis of Statistics Assessment and Review Instrument
LTFU: Lost to follow up from art
OI/s: Opportunistic infection(s)
*OR*: Odds ratio
PLHIV: People living with HIV
SSA: Sub-Saharan Africa
TB: Tuberculosis
UNAIDS: The Joint United Nations Program on HIV/AIDS
WHO: World Health Organization
