# Supplementary material for: Predictors of lost to follow up from antiretroviral therapy among adults in sub-Saharan Africa: a systematic review and meta-analysis
Source: Infect Dis Poverty. 2021 Mar 20;10:33. doi: 10.1186/s40249-021-00822-7 (PMC7981932; doi:10.1186/s40249-021-00822-7)
Supplement: Supplementary file 1 — Additional file 1: Table S1. Full searching strategy by databases. S1 doc. JBI Critical Appraisal instruments. S2 doc. JBI Data extraction instruments. S3 doc. PRISMA Checklist of items to include when reporting a systematic review or meta-analysis. Table S2. Characteristics of included articles (n = 30). Table S3. Assessment of methodological quality (n = 30). Table S4. Risk of Bias Assessment within the studies (n = 30) [file 40249_2021_822_MOESM1_ESM.docx]

# **Additional files**

## **Table S1: Full searching strategy by databases**

***Medline searching strategy****

| 1 | (“Lost to follow-up” or “LTFU” or discontinuation or attrition or retention or defaulting or “discontinuation rate” or “attrition rate” or “retention rate” or defaulting rate”).tw. |
| --- | --- |
| 2 | (Predictors or factors or determinants).tw. |
| 3 | (“Antiretroviral therap*” “ART” or “Highly active antiretroviral therap*” or “HAART” or “Human Immuno deficiency virus” or “HIV” or “AIDS”) ).tw. |
| 4 | (“Sub-Saharan Africa” or Africa).tw. |
| 5 | 1 and 2 and 3 and 4; |

*MeSH terms to be added during searching

***PubMed searching strategy****

| 1 | (“lost to follow up” OR “ltfu” OR discontinuation OR retention OR attrition OR adherence OR retention OR attrition OR compliance OR “ltfu” OR discontinuation OR defaulting OR ((“loss to follow-up”) OR (“loss to follow up”) OR (“lost to follow-up”) OR (“lost to follow up”) OR (“loss-to-follow-up”) OR (“lost-to-follow-up”) OR (“loss to retention”) OR (“lost to retention”) OR (“treatment initiation”) OR (“retention”) OR (“retain*”) OR (“attrition”)) OR ((“adher*”) OR (“complian*”) OR (“comply”) OR (“complied”) OR (“noncomplian*”) OR (“non-complian*”) OR (“non-adher*”) OR (“nonadher”))) |
| --- | --- |
| 2 | (Predictors OR Factors OR Determinants) |
| 3 | (“Antiretroviral therapy” OR “art” OR “haart” OR “Highly active antiretroviral therapy” OR “human Immuno deficiency virus” OR “hiv” OR “aids”)  (“hiv” OR “art” OR “triple therapy” OR “haart” OR “aids” **OR** "HIV Infections"[Mesh] OR "HIV" [MeSH] OR “human immunodeficiency virus”[tiab] OR “human immuno deficiency virus”[tiab] OR “human immune deficiency virus”[tiab] OR “human immunedeficiency virus”[tiab] OR “aids”[tiab] OR “acquired immunodeficiency syndrome”[tiab] OR “acquired immunodeficiency syndromes”[tiab] OR “acquired immuno deficiency syndrome”[tiab] OR “acquired immuno deficiency syndromes”[tiab] OR “acquired immune deficiency syndrome”[tiab] OR “acquired immune deficiency syndromes”[tiab] OR “acquired immunedeficiency syndrome”[tiab] OR “acquired immunedeficiency syndromes”[tiab] |
| 4 | (Africa OR “Sub Saharan Africa”) |
| 5 | (1 AND 2 AND 3AND 4) NOT Medline[sb])” LIMITED to English, adult, time(2002-2019), AIDS |

* MeSH terms to be added during searching

***Web of Science searching strategy***

| 1 | TS= (“Lost to follow-up” or “LTFU” or discontinuation or attrition or retention or defaulting or “discontinuation rate” or “attrition rate” or “retention rate” or “defaulting rate”) |
| --- | --- |
| 2 | TS= (Predictors or factors or determinants) |
| 3 | TS= (“Anti-retroviral therap*” “ART” or “Highly active antiretroviral therap*” or “HAART” or “Human Immuno deficiency virus” or “HIV” or “AIDS”) |
| 4 | TS= (“Sub-Saharan Africa” or Africa) |
| 5 | 1 AND 2 AND 3 AND 4; Limited to language (English), age (adult), time(2002-2019), subject (AIDS) |

***Scopus searching strategy***

| 1 | ALL (“lost to follow up” OR “LTFU” OR discontinuation OR attrition OR retention OR defaulting OR “discontinuation rate” OR “attrition rate” OR “retention rate” OR “defaulting rate”) |
| --- | --- |
| 2 | ALL (predictors OR factors OR determinants) |
| 3 | ALL (“Ant-retroviral therap*” OR “ART” OR “HAART” OR “Highly active antiretroviral therap*” OR “human Immuno deficiency virus” OR “HIV” OR “AIDS”)” LIMITED to English |
| 4 | ALL(Africa OR “Sub-Saharan Africa”) |
| 5 | 1 AND 2 AND 3 AND 4; Limited to language (English), age (adult), time(2002-2019), subject (AIDS) |

***CINAHL Searching strategy****

| S1 | Lost to follow-up” or “LTFU” or discontinuation or attrition or retention or defaulting or “discontinuation rate” or “attrition rate” or “retention rate” or “defaulting rate” |
| --- | --- |
| S2 | Predictors or factors or determinants |
| S3 | “Anti-retroviral therap*” “ART” or “Highly active antiretroviral therap*” or “HAART” or “Human Immuno deficiency virus” or “HIV” or “AIDS”) |
| S4 | “Sub-Saharan Africa” or Africa |
| S5 | S1 AND S2 AND S3 AND S4; Limited to language (English), age (adult), time(2002-2019), subject (AIDS) |

*MH words to be added during searching

**S1 doc: JBI Critical Appraisal instruments**


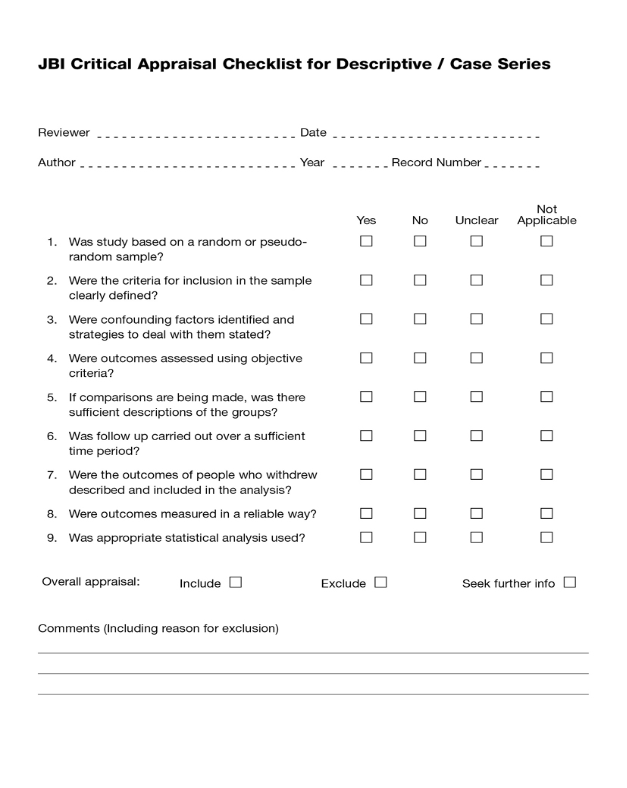


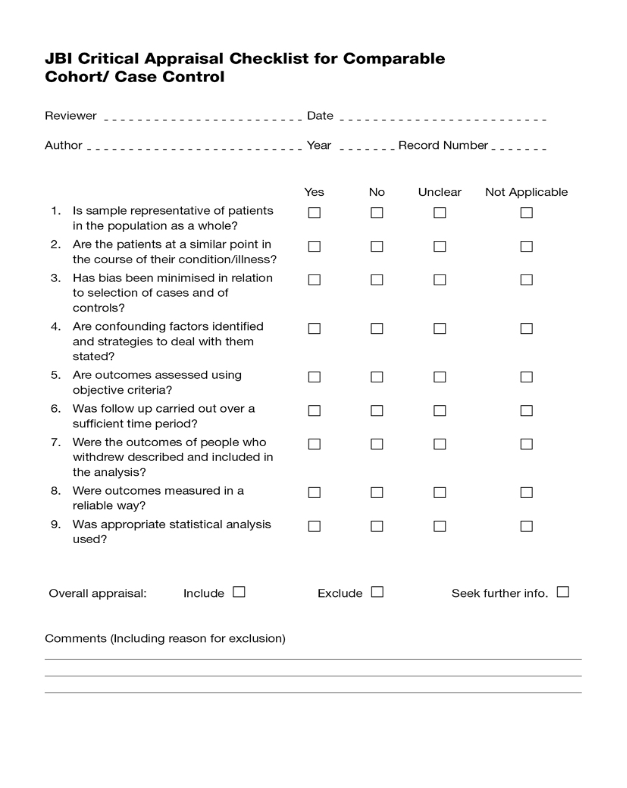


## **S2 doc: JBI Data extraction instruments**


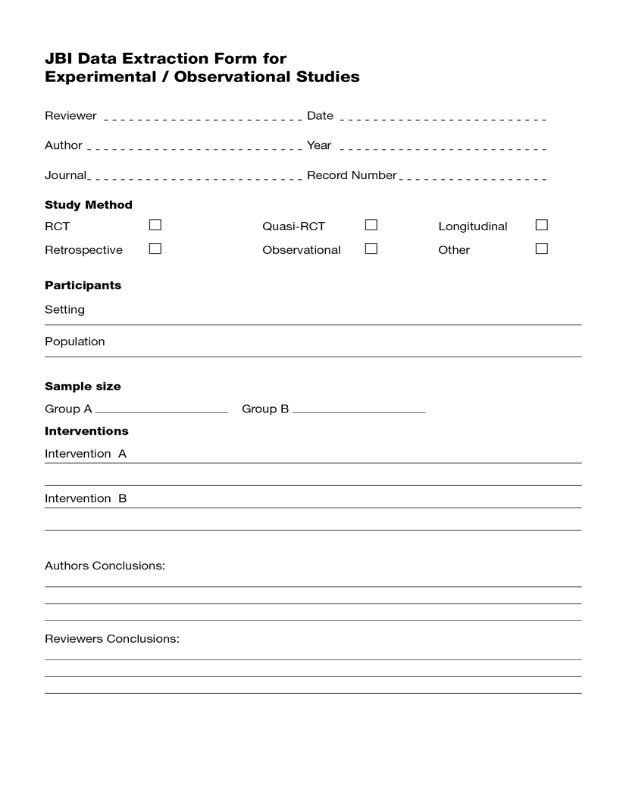


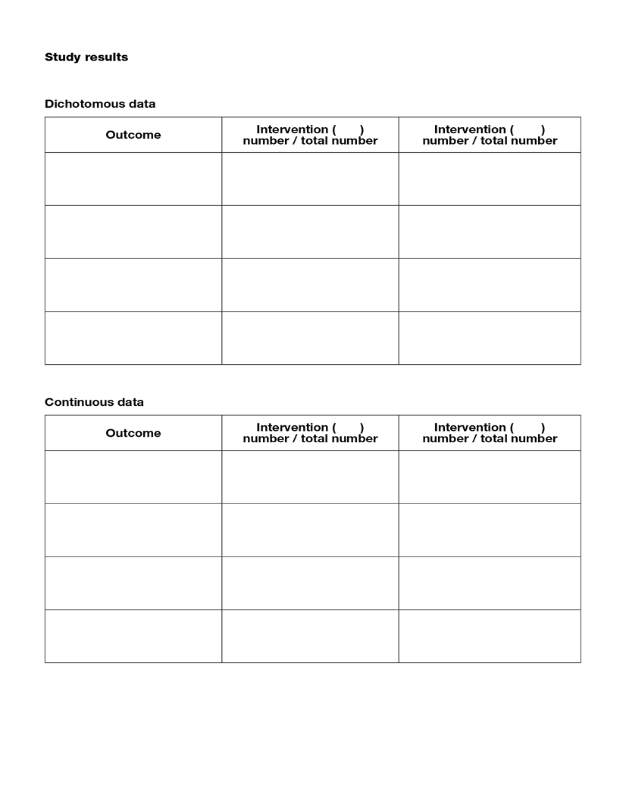


**S3 doc: PRISMA Checklist of items to include when reporting a systematic review or meta-analysis** [Adapted from *Preferred reporting items for systematic review and meta-analysis protocols (PRISMA-P) 2015 statement* ***by* Moher D et al, 2015**]

| **Section/topic** | **#** | **Checklist item** | **Information reported** |  |  |
| --- | --- | --- | --- | --- | --- |
|  |  |  | **Yes** | **No** | **Page number(s)** |
| **ADMINISTRATIVE INFORMATION** |  |  |  |  |  |
| **Title** |  |  |  |  |  |
| Identification | 1a | Identify the report as a protocol of a systematic review | √ |  | 5 |
| Update | 1b | If the protocol is for an update of a previous systematic review, identify as such |  | √ |  |
| **Registration** | 2 | If registered, provide the name of the registry (e.g., PROSPERO) and registration number in the Abstract | √ |  | CRD42018114418 |
| **Authors** |  |  |  |  |  |
| Contact | 3a | Provide name, institutional affiliation, and e-mail address of all protocol authors; provide physical mailing address of corresponding author | √ |  | 1 |
| Contributions | 3b | Describe contributions of protocol authors and identify the guarantor of the review | √ |  | 29 |
| **Amendments** | 4 | If the protocol represents an amendment of a previously completed or published protocol, identify as such and list changes; otherwise, state plan for documenting important protocol amendments | √ |  |  |
| **Support** |  |  |  |  |  |
| Sources | 5a | Indicate sources of financial or other support for the review | √ |  | 29 |
| Sponsor | 5b | Provide name for the review funder and/or sponsor |  |  | 29 |
| Role of sponsor/funder | 5c | Describe roles of funder(s), sponsor(s), and/or institution(s), if any, in developing the protocol |  |  | 29 |
| **ABSTRACT** Structured summary | 6 | Provide a structured summary: background, objectives, methods, results, conclusions and implications of key findings | √ |  | 2-3 |
| **INTRODUCTION** |  |  |  |  |  |
| **Rationale** | 8 | Describe the rationale for the review in the context of what is already known | √ |  | 3-5 |
| **Objectives** | 8 | Provide an explicit statement of the question(s) the review will address with reference to participants, interventions, comparators, and outcomes (PICO) | √ |  | 4-5 |
| **METHODS** |  |  |  |  |  |
| **Eligibility criteria** | 9 | Specify the study characteristics (e.g., PICO, study design, setting, time frame) and report characteristics (e.g., years considered, language, publication status) to be used as criteria for eligibility for the review | √ |  | 5 |
| **Information sources** | 10 | Describe all intended information sources (e.g., electronic databases, contact with study authors, trial registers, or other grey literature sources) with planned dates of coverage | √ |  | 6 |
| **Search strategy** | 11 | Present draft of search strategy to be used for at least one electronic database, including planned limits, such that it could be repeated | √ |  | 6-7 |
| ***STUDY RECORDS*** |  |  |  |  |  |
| Data management | 12a | Describe the mechanism(s) that will be used to manage records and data throughout the review | √ |  | 6-7 |
| Selection process | 12b | State the process that will be used for selecting studies (e.g., two independent reviewers) through each phase of the review (i.e., screening, eligibility, and inclusion in meta-analysis) | √ |  | 6-7 |
| Data collection process | 12c | Describe planned method of extracting data from reports (e.g., piloting forms, done independently, in duplicate), any processes for obtaining and confirming data from investigators | √ |  | 7 |
| **Data items** | 13 | List and define all variables for which data will be sought (e.g., PICO items, funding sources), any pre-planned data assumptions and simplifications | √ |  | 5-7 |
| **Outcomes and prioritization** | 14 | List and define all outcomes for which data will be sought, including prioritization of main and additional outcomes, with rationale | √ |  | 5 |
| **Risk of bias in individual studies** | 15 | Describe anticipated methods for assessing risk of bias of individual studies, including whether this will be done at the outcome or study level, or both; state how this information will be used in data synthesis | √ |  | 9 |
| ***DATA*** |  |  |  |  |  |
| **Synthesis** | 16a | Describe criteria under which study data will be quantitatively synthesized | √ |  | 9 |
|  | 16b | If data are appropriate for quantitative synthesis, describe planned summary measures, methods of handling data, and methods of combining data from studies, including any planned exploration of consistency (e.g., *I* ^2^, Kendall’s tau) | √ |  | 9 |
|  | 16c | Describe any proposed additional analyses (e.g., sensitivity or subgroup analyses, meta-regression) | √ |  | 9 |
|  | 16d | If quantitative synthesis is not appropriate, describe the type of summary planned | √ |  | 9 |
| **Meta-bias(es)** | 17 | Specify any planned assessment of meta-bias(es) (e.g., publication bias across studies, selective reporting within studies) | √ |  | 9 |
| **Confidence in cumulative evidence** | 18 | Describe how the strength of the body of evidence will be assessed (e.g., GRADE) | √ |  | 9 |
| **RESULTS** | | | | | |
| Study selection | 19 | Give numbers of studies screened, assessed for eligibility, and included in the review, with reasons for exclusions at each stage, ideally with a flow diagram. | √ |  | 6-7 |
| Study characteristics | 20 | For each study, present characteristics for which data were extracted (e.g., study size, PICOS, follow-up period) and provide the citations. | √ |  | 8-10 |
| Risk of bias within studies | 21 | Present data on risk of bias of each study and, if available, any outcome level assessment (see item 12). | √ |  | 10 |
| Results of individual studies | 22 | For all outcomes considered (benefits or harms), present, for each study: (a) simple summary data for each intervention group (b) effect estimates and confidence intervals, ideally with a forest plot. | √ |  | 13-16 |
| Synthesis of results | 23 | Present results of each meta-analysis done, including confidence intervals and measures of consistency. | √ |  | 13-25 |
| Risk of bias across studies | 24 | Present results of any assessment of risk of bias across studies (see Item 15). | √ |  | Supportive doc table 2 |
| Additional analysis | 25 | Give results of additional analyses, if done (e.g., sensitivity or subgroup analyses, meta-regression [see Item 16]). |  | √ |  |
| **DISCUSSION** | | | | | |
| Summary of evidence | 26 | Summarize the main findings including the strength of evidence for each main outcome; consider their relevance to key groups (e.g., healthcare providers, users, and policy makers). | √ |  | 15-18 |
| Limitations | 27 | Discuss limitations at study and outcome level (e.g., risk of bias), and at review-level (e.g., incomplete retrieval of identified research, reporting bias). | √ |  | 17-18 |
| Conclusions | 28 | Provide a general interpretation of the results in the context of other evidence, and implications for future research. | √ |  | 18 |
| **FUNDING** | | | | | |
| Funding | 29 | Describe sources of funding for the systematic review and other support (e.g., supply of data); role of funders for the systematic review. | √ |  | 19 |

**Table S2: Characteristics of included articles (n=30)**

| **Author** | **Year** | **Sample size (n)** | **Study design** | **Outcome of interest** | **Measurement** | **Setting** | **Summary** |
| --- | --- | --- | --- | --- | --- | --- | --- |
| Ahonkhai et al.[38] | 2012 | 11,397 | Retrospective cohort | LTFU | Missing all follow-up visits in the first 12 months on ART. | South Africa | Enrolment in later calendar year (2008 Vs 2004) (AOR 0.49, 95%CI 0.39–0.62) and being female (AOR 0.82; 95%CI 0.73–0.91) had lower risk of LTFU |
| Akilimali et al. [39] | 2017 | 844 | Retrospective Cohort | LTFU | Stop attending ART clinic for more than 3 months and had not yet been classified as dead' or `transferred-out' | Goma, Democratic Republic of Congo | Patients who did not disclosed their HIV status (AHR 2.28, 95% CI 1.46±2.29), rural residents (AHR 1.97, 95% CI 1.02±3.77), and being more educated (AHR 1.60, 95% CI 1.02±2.53) were at higher risk of being lost from treatment. |
| Asefa et al.[25] | 2013 | 236 | Case control | Defaulting | HIV positive patient who had been enrolled on ART treatment and who failed to return for two or months | Western Ethiopia, Ethiopia | Rural residence (AOR=4.1; 95%CI 1.86 to 9.42), no income (AOR=13.9; 95%CI 4.23 to 45.99), having psychiatric illness (AOR=4.7; 95%CI 1.65 to 13.35), having discordant partner (AOR=5.1; 95%CI 1.59 to 16.63), having partner with unknown HIV status   (AOR=2.8; 95% CI 1.23 to 6.50) and having a concern for stigma (AOR=8.3; 95% CI 2.88 to 23.83) were predictors for exit from treatment |
| Assemie et al.[40] | 2018 | 602 | Retrospective Cohort | LTFU | Patients not taking ART refill for a period of 3 months or longer from the last attendance for refill and not yet classified as ‘died’ or ‘transferred-out’ | Northwest Ethiopia,  Ethiopia | Being young (15–28 years, AHR = 0.44; 95% CI 0.24–0.83), advanced WHO clinical stage (stage IV, AHR = 2.09; 95% CI 1.02–3.13); and receiving isoniazid preventive therapy (AHR = 0.11; 95% CI 0.06–0.18) were independent significant predictors of lost to follow up |
| Berheto et al. [22] | 2014 | 2133 | Retrospective cohort | LTFU | Not taking ART refill for a period of three months or longer from the last attendance and not yet classified as ‘dead’ or ‘transferred-out’ | Mizan, Southwest Ethiopia | Patients with regimen substitution (AHR=5.2, 95% CI: 3.6-7.3), non-isoniazid (INH) prophylaxis (AHR=3.7, 95% CI: 2.3-6.2), adolescent (HR=2.1, 95% CI: 1.3-3.4), and had a baseline CD4 count < 200 cells/mm3 (AHR=1.7, 95% CIs: 1.3-2.2) were at higher risk of LTFU. WHO clinical stage 3 (AHR=0.6, 95% CIs: 0.4-0.9) and 4 (AHR=0.8, 95% CI: 0.6-1.0) patients at entry were less likely to be LTFU than clinical stage 1 patients. |
| Bucciardini et al.[10] | 2015 | 563 | Prospective  Cohort | LTFU | Patients who lost scheduled visit to the same ART clinic more than three months after the last visit.  Or  Patients known to have discontinued ART for any reasons | Tigray, Northern Ethiopia | Low level Health Facility (AHR 2.99, 95% CI: 2.77–3.23), Active TB (HR 1.72, 95% CI: 1.23–2.41) and male gender (AHR 1.64, 95% CI: 1.10–2.56) were the predictors of attrition. |
| Deribe et al. [60] | 2008 | 1270 | Case control study | Defaulting | An individual who had been on ART treatment and who had not been seen for the last 2 months | Jimma, Ethiopia | Taking hard drugs (cocaine, cannabis and IV drugs) (AOR = 0.02, 95%CI: 0.003±0.17), excessive alcohol consumption (AOR = 6, 95%CI: 3.3±11.1), being bedridden (AOR = 5.7, 95% CI: 1.6±20.2), rural residence (AOR = 2.2, 95%CI: 1.4±3.5) and having sero-discordant (AOR = 3.5, 95%CI: 1.1±11.1) or unknown HIV status partner (AOR = 1.7, 95%CI: 1.02 = 2.9) were associated with ART defaulting. |
| Dessalegn et al.[5] | 2015 | 727 | Case control | LTFU | Patients who had missed two or more clinical appointments since the last scheduled visit | Wukro, Northern Ethiopia | Presence of bereavement concern (AOR=0.1, 95%CI: 0.01-0.3), not being provided with Isoniazid prophylaxis (AOR=3.04, 95%CI: 1.3-7.3), and presence of side effects (AOR=12.3, 95%CI: 4.9-31.4) were found determinants of lost to follow up |
| Djarma et al. [41] | 2014 | 509 | Retrospective cohort | LTFU | A time delay of 3 months since the last scheduled visit. | N’Djamen, Chad | Stable free access to HAART (72.5 vs. 10%; p,0.001) was associated with decreasing lost from care. On the contrary, living outside the town (24.2% vs 9.2%; p¼0.001) was associated with increasing risk of LTFU |
| Eguzo et al. [42] | 2015 | 1256 | Retrospective Cohort | LTFU | A patient who missed an appointment for more than 6 months after the last visit | Aba­Nigeria, Nigeria | Early calendar year enrollment (2008 Vs 2010-2013) (HR 3.1, 95% CI 1.16–8.17; P = 0.02) and ((2009 Vs 2010-2013) (AHR 2.69 95% CI 1.05–6.88, P = 0.04)) were more likely to be LTFU |
| Fatti et al. [43] | 2010 | 29203 | Retrospective cohort | LTFU | Absence from the clinic for three or more months after the last missed appointment date and not known to have died or transferred treatment program or place. | South Africa | Being male (AHR 1.20 95% CI 1.09–1.33 P 0.001) and enrolled in later years (AHR 6.18, 95% CI: 4.30–8.89; P< 0.001) had elevated risks of LTFU. On the other hand, Older age was inversely related to the risk of LTFU (AHR 0.89 95% CI 0.84–0.94, P <0.001) |
| Gesesew et al. [44] | 2017 | 4900 | Retrospective Cohort | LTFU | Patients who failed to pick up their medications for at least 3 months but had not yet been classified as dead or transferred out | Jimma, Southwest Ethiopia | Being female (AOR = 2.1, 95%CI: 1.7±2.8), developing immunological failure (AOR = 2.3, 1.9±8.2), having tuberculosis co-infection (AOR=1.5, 1.1±2.1) and having no history of HIV testing (AOR = 1.8, 1.4±2.9) were more likely to discontinue care. |
| Gezae et al. [45] | 2019 | 305 | Retrospective Cohort | LTFU | Patients who failed to collect ART drugs for at least three consecutive months and not recorded as dead, alive, transfer, drop out or stop | Mekelle, Ethiopia | Low Hemoglobin level (≤ 11.0 g/dl, AHR = 2.660; 95%CI: 1.459–4.848), and any history of OI/s (AHR = 3.795; 95%CI: 1.165–12.364) were associated with LTFU. Whereas, adverse drug events (AHR = 0.451; 95%CI: 0.216–0.941), TB treatment completion (AHR = 0.121; 95% CI: 0.057–0.254), and being on Isoniazid Preventive Therapy (IPT) (AHR = 0.085; 95%CI: 0.012–0.628) had protective effect against LTFU. |
| Ibiloye et al. [46] | 2018 | 710 | Retrospective cohort | LTFU | Missing treatment for more than 2 months since the last appointment. | Nasarawa state, Nigeria. | Patients having no formal education (AHR 1.8; 95% CI 1.3–2.6, P= 0.001) and unemployed (AHR 1.8; 95% CI 1.2–2.6, p= 0.001) were more likely to get lost from the care |
| Kaplan et al. [48] | 2017 | 39,884 | Retrospective Cohort | Disengagement | A patient stopping accessing care in an ART clinic for *>*6 months after his or her last visit date. | Cape Town, South Africa | Recent lower CD4 count(CD4 200±350 HR 2.03; 95% CI 1.91±2.15; CD4 50±200 HR 3.07; 95% CI 2.84±3.31; CD4 *<*50 HR 3.34; 95% CI 2.92±3.83, all relative to CD4 *>* 350), use of d4T (stavudine) at last visit (AHR 1.72; 95% CI 1.57±1.89), and initiating ART during pregnancy (AHR 1.58; 95% CI 1.47±1.69) were determinants of lost from care. |
| Katz et al. [47] | 2017 | 4025 | Retrospective Cohort | LTFU | Break off from treatment within the first 4 months on ART | Cape Town, South Africa | Linking to ART in later calendar year (AOR= 1.30, 95% CI 1.09±1.55), lower CD4 count at ART initiation (< 200; AOR = 1.34, 95% CI 1.06±1.67). |
| Kiguba et al. [61] | 2007 | 686 | Cross sectional | Discontinuation | Simultaneous stopping of all antiretrovirals for at least 1 month | Kampala, Uganda. | HAART experience before starting the current regimen (AOR = 3.70, 95% CI: 2.13 to 6.25), use of alternative medicines (AOR = 2.18, 95% CI: 1.06 to 4.47), history of hospitalization (AOR = 2.36, 95% CI: 1.32 to 4.20), and 1 year or less on ART (OR = 11.11, 95% CI: 5.00 to 25.00) were the determinants of discontinuation. |
| Mberi et al. [20] | 2015 | 595 | Retrospective cohort | LTFU | A patient who had been failed to return to the health facility for more than six months since their last expected date of return | South Africa | Having no regular partner (AHR: 2.9, 95 % CI:1.19-6.97, p = 0.019), owning private business (AHR: 13.9, 95 % CI:2.81 - 69.06, p = 0.001), higher baseline CD4 count (AHR: 3.8, 95 % CI: 1.85-7.85, p < 0.001), virologic failure in the last visit (AHR: 3.6, 95 % CI:1.98 - 6.52, p < 0.001) and a last known advanced WHO treatment stage (AHR: 2.0, 95 % CI:1.22-3.27, p = 0.006) had a higher risk of LTFU. Patients with a history of ART adverse event had a lower risk (AHR: 0.6, 95 % CI: 0.38 - 0.99, p = 0.044) of becoming LTFU than those who had not. |
| Megerso et al. [59] | 2016 | 1248 | Case–control study | LTFU | Individual patients who were registered as LTFU during the 12 months prior to the survey | Oromia, Ethiopia | Age 15–24 years (AOR 19.82, 95% CI: 6.80, 57.73); day laborers (AOR, 5.36; 95% CI: 3.23, 8.89), rural residents (AOR, 2.35; 95% CI: 1.45, 3.89), World Health Organization clinical stage 4 (AOR, 2.29; 95% CI: 1.45, 3.62), baseline CD4 count <350 cells/mL (AOR, 2.06; 95% CI: 1.36, 3.13), and suboptimal adherence to ART (AOR, 7.42; 95% CI: 1.87, 29.41), were factors which increased the risk of loss to follow-up in ART. |
| Mekonnen et al. [50] | 2019 | 569 | Retrospective cohort | LTFU | Missing from the clinic for at least three months after the last missed appointment but not transferred out from the facility to another facility or died. | Gondor, North West Ethiopia | Being underweight (AHR, 1.52, 95% CI 1.01–2.28), unemployed (AHR, 2.22, 95% CI 1.2–4.11), substance abuser (AHR, 1.84 95% CI 1.19–2.86), having sub-optimal adherence (AHR 6.33, 95% CI (3.90–10.26)), not received isoniazid prophylaxis (AHR 2.47, 95% CI (1.36–4.48)), ambulatory functional status (AHR 1.94, 95% CI (1.23–3.06)), opportunistic infections (AHR, 1.74 95% CI 1.11–2.72), lower CD4 count (AHR 0.58, 95% CI (0.38–0.88)) were found to be significant predictors of lost to follow up from ART service |
| Melaku et al. [13] | 2015 | 53,300 | Retrospective longitudinal | LTFU | Failure to visit ART clinic for more than 6 months and was not recorded as dead or transferred | Ethiopia | Being male (Female Vs Male AHR 0.73, 95% CI: 0.70-0.76), Younger age (AHR (50+ vs. 15–24): 0.67, 95% CI: 0.54-0.81; (40–49 vs. 15–24): 0.67, 95% CI: 0.60-0.75; (25–39 vs. 15–24): 0.77, 95% CI: 0.72-0.83), being unmarried ((Married/Living together Vs Never Married AHR 0.67 0.63-0.71),(Separated/Divorced Vs Never Married AHR 0.90 0.84-0.96), (Widowed Vs Never Married AHR 0.78 0.71-0.86)), No formal education((Primary Vs No Education AHR 0.71 0.66-0.75), (Secondary Vs No Education AHR 0.59 0.53-0.66), (Tertiary Vs No Education AHR 0.48 0.42-0.55)), low CD4+ cell count ((<100 Vs 350+ AHR 1.43 1.20-1.71), and advanced WHO clinical stage ((Stage 3 Vs Stage 1 AHR 1.19 1.07-1.32), Stage 4 Vs Stage 1 AHR 1.60 1.38-1.85)), Later calendar year ART initiation (2010–11 vs 2006–07 AHR 1.27, 95% CI:1.15-1.40) were found to be at high risk of LTFU from ART. |
| Meloni et al. [52] | 2016 | 51953 | Retrospective cohort | LTFU | Patients who had failed to visit the clinic for more than two months since the last scheduled pick-up date. | Nigeria | Being young age (<30 years Vs p<0.001), unemployed(p<0.001), having no formal education (p<0.001), being unmarried (p<0.001), WHO clinical stage (p<0.001), having detectable viral load (p<0.001), and lower CD4+ cell counts (p<0.001) were determinants of lost from care. |
| Mugisha et al. [53] | 2014 | 31,033 | Retrospective cohort | LTFU | Failed to be active in ART care for 6 months and was not registered as death, withdrawal or transfer to another health facility. | Kigali City and western  Rwanda, Rwanda | Male (AHR) 1.4, 95%CI 1.2–1.7) and younger age (AHR 1.4, 95%CI 1.2–1.7) patients were more likely to be LTF. Being married (AHR 0.6, 95%CI 0.4–0.9), and having lower base line CD4 count (AHR 0.64, 95%CI 0.4–0.9) were protective against LTF in ART patients. |
| Ochieng-Ooko et al.[54] | 2010 | 50, 275 | Retrospective Cohort | LTFU | A patient who failed to collect his/her medication (ART) for more than 3 months | Kenya | Being male gender (AOR=1.24, 95%CI: 1.15-1.33), living far away from clinic (≥ 1 hour travel time) (AOR=.1.11, 95%CI: 1.04-1,19) and advanced WHO clinical stage-(3 or 4) (AOR=1.30, 95%CI: 1.21-1.40) were linked with lost to follow-up. In contrast, being elderly (AOR=0.59, 95%CI: 0.55-.0.64), sharing HIV+ status (AOR=0.91, 95%CI: 0.85-0.98) and enrolling in a later time period (AOR=0.85, 95%CI: 0.79-0.90) were all associated with a lower risk of LTFU. |
| Seifu et al. [55] | 2018 | 1439 | Retrospective Cohort | LTFU | Stop accessing ART treatment for more than 3 months and not yet classified as dead or transferred-out | Jigjiga, Ethiopia | Being male (HR: 2.1 CI;1.3–3.4, p= 0.034), no next appointment record [AHR: 1.2, 95% CI; (1.12–1.36), p= 0.000*) and failure to disclose HIV status to any one (AHR: 2.8, 95% CI; (2.22–5.23), p= 0.034*) were predictors of LTFU. |
| Tadesse et al. [12] | 2014 | 520 | Retrospective cohort | LTFU | Patients who had missed one or more clinical appointments | Axum, Northern Ethiopia | Being smear positive pulmonary Tb (AHR=2.05, 95% CI: 1.02, 4.12), male gender (AHR=2.73, 95%CI: 1.31, 5.66), regiment AZT-3TC-NVP (AHR=3.47, 95%CI: 1.02,11.83) and weight ≥60kg (AHR= 0.24, 95% CI: 0.06,0.96). |
| Teshome et al. [11] | 2015 | 1173 | Retrospective cohort | LTFU | Missing from ART clinic for more than 3 months after the last appointment date | South Ethiopia | Being with in the normal body weight range (AHR=0.6, 95%CI: 0.4-0.9), advanced disease stage (AHR=1.4, 95%CI: 1.02-1.9), having no isoniazid prophylaxis (AHR=1.9, 95%CI=1.1-3.2), being in the adult age group (AHR=0.6, 95%CI: 0.4-0.8), serving in lower level health facility (AHR=0.7, 95% CI: 0.5-0.9), and higher educational status (AHR=0.6, 95%CI: 0.4-0.7) were predictors of LTFU. |
| Tweya et al. [56] | 2017 | 37,378 | Retrospective cohort | LTFU | A patient who did not return to the ART clinic for more than 2 months | Lilongwe, Malawi | LTFU was lower among those aged > 50 years when compared to younger patients aged 25±39 years (6.3 (95% CI 6.1±6.5), 4.5 (95% CI 4.2±4.7), and 5.6 (95% CI 5.1± 6.1). |
| Van Cutsem et al. [58] | 2011 | 6411 | Prospective cohort | LTFU | No clinic visits for at least 6 months and not classified as died or transferred out. | South Africa | Being young age (AHR=0.87, 95% CI 0.78–0.98, P=0.017), pregnancy (AHR 1.85, p = 0.072) and increasing calendar year (p,0.001) were remained associated with true LTF |
| Wang et al. [57] | 2011 | 925 | Retrospective Cohort | LTFU | Missing scheduled appointment within 6 months after ART initiation  Or  No documented information in the database on a patient returning for a 6-month visit. | Tapologo, South Africa | Being young age (AHR 2.14, 95% CI 1.05 - 4.38), pregnancy (AHR 3.75, 95% CI 1.53- 9.16) were predictors of LTFU. Those who were immune suppressed and pregnant women with lower baseline CD4 counts (≤200 cells/μl) had 6.06 times the hazard (95% CI 2.20 - 16.71) of LTFU at 6 months compared to men |

AHR: adjusted hazard ratio; AOR= adjusted odds ratio; CD4: cluster of differentiation 4; CI: confidence interval; HAART: highly active antiretroviral therapy; INH: non-isoniazid LTFU: lost to follow up; TB: tuberculosis; WHO: World Health Organization

**S3: Assessment of methodological quality (n=30)**

| Authors | Q1 | Q2 | Q3 | Q4 | Q5 | Q6 | Q7 | Q8 | Q9 | % |
| --- | --- | --- | --- | --- | --- | --- | --- | --- | --- | --- |
| Ahonkhai et | Y | Y | Y | Y | Y | Y | N | Y | Y | 89 |
| Akilimali et al | Y | Y | Y | Y | Y | Y | NA | Y | Y | 100 |
| Asefa et al. | Y | Y | Y | Y | Y | NA | N | Y | Y | 88 |
| Assemie et al. | Y | Y | Y | Y | Y | Y | Y | Y | Y | 100 |
| Berheto et al. | Y | Y | Y | Y | Y | Y | NA | Y | Y | 100 |
| Braitsten et al. | Y AAA | Y | Y | Y | Y | Y | Y | Y | Y | 100 |
| Bucciardini et al. | Y | Y | Y | Y | Y | Y | N | Y | Y | 89 |
| Deribe et al. | Y | Y | Y | Y | Y | NA | N | Y | Y | 88 |
| Dessalegn et al. | Y | Y | Y | Y | Y | NA | NA | Y | Y | 100 |
| Djarma et al. | Y | Y | Y | Y | Y | Y | N | Y | Y | 89 |
| Eguzo et al. | Y | Y | Y | Y | Y | Y | Y | Y | Y | 100 |
| Fatti et al. | Y | Y | Y | Y | Y | Y | Y | Y | Y | 100 |
| Gesesew et al. | Y | Y | Y | Y | Y | Y | N | Y | Y | 89 |
| Gezae et al | Y | Y | Y | Y | Y | Y | Y | Y | Y | 100 |
| Ibiloye et al. | Y | Y | Y | Y | Y | Y | Y | Y | Y | 100 |
| Kaplan et al. | Y | Y | Y | Y | Y | Y | Y | Y | Y | 100 |
| Katz et al. | Y | Y | Y | Y | Y | Y | N | Y | Y | 89 |
| Kiguba et al. | Y | Y | Y | Y | Y | NA | NA | Y | Y | 100 |
| Mberi et al. | Y | Y | Y | Y | Y | Y | Y | Y | Y | 100 |
| Megerso et al. | Y | Y | Y | Y | Y | NA | NA | Y | Y | 100 |
| Mekonnen et al. | Y | Y | Y | Y | Y | Y | Y | Y | Y | 100 |
| Melaku et al. | Y | Y | Y | Y | Y | Y | NA | Y | Y | 100 |
| Meloni et al. | Y | Y | Y | Y | Y | Y | Y | Y | Y | 100 |
| Mugisha et al. | Y | Y | Y | Y | Y | Y | Y | Y | Y | 100 |
| Seifu et al. | Y | Y | Y | Y | Y | Y | Y | Y | Y | 100 |
| Tadesse et al. | Y | Y | Y | Y | Y | Y | NA | Y | Y | 100 |
| Teshome et al. | Y | Y | Y | Y | Y | Y | NA | Y | Y | 100 |
| Tweya et al. | Y | Y | Y | Y | Y | Y | Y | Y | Y | 100 |
| Van Cutsem et al. | Y | Y | Y | Y | Y | Y | Y | Y | Y | 100 |
| Wang et al. | Y | Y | Y | Y | Y | Y | Y | Y | Y | 100 |

Q= Question Y=Yes; N= No; NA= Not applicable

**S4: Risk of Bias Assessment within the studies (n=30)**

| Study | Random Sequence Generation (Selection bias) | Allocation Concealment (Selection bias) | Blinding of Participants and personnel (Performance bias) | Blinding of outcome Assessment (Detection bias) | Incomplete Outcome Data (attrition bias) | Selective reporting (Reporting bias) | Other |
| --- | --- | --- | --- | --- | --- | --- | --- |
| Ahonkhai AA et | Unclear risk^a^ | Unclear risk^a^ | Unclear risk | Low risk | Low risk | Low risk | Low risk |
| Akilimali et al | Unclear risk^a^ | Unclear risk^a^ | Unclear risk | Low risk | Low risk | Low risk | Low risk |
| Asefa et al. | Unclear risk^a^ | Unclear riska | Unclear risk | Low risk | Low risk | Low risk | Low risk |
| Assemie et al. | Unclear risk^a^ | Unclear risk^a^ | Unclear risk | Low risk | Low risk | Low risk | Low risk |
| Berheto et al. | Unclear risk^a^ | Unclear risk^a^ | Unclear risk | Low risk | Low risk | Low risk | Low risk |
| Braitsten P et al. | Unclear risk^a^ | Unclear risk^a^ | Unclear risk | Low risk | Low risk | Low risk | Low risk |
| Bucciardini et al. | Unclear risk^a^ | Unclear risk^a^ | Unclear risk | Low risk | Low risk | Low risk | Low risk |
| Deribe et al. | Unclear risk^a^ | Unclear risk^a^ | Unclear risk | Low risk | Low risk | Low risk | Low risk |
| Dessalegn et al. | Unclear risk^a^ | Unclear risk^a^ | Unclear risk | Low risk | Low risk | Low risk | Low risk |
| Djarma et al. | Unclear risk^a^ | Unclear risk^a^ | Unclear risk | Low risk | Low risk | Low risk | Low risk |
| Eguzo et al. | Unclear risk^a^ | Unclear risk^a^ | Unclear risk | Low risk | Low risk | Low risk | Low risk |
| Fatti et al. | Unclear risk^a^ | Unclear risk^a^ | Unclear risk | Low risk | Low risk | Low risk | Low risk |
| Gesesew et al. | Unclear risk^a^ | Unclear risk^a^ | Unclear risk | Low risk | Low risk | Low risk | Low risk |
| Gezae et al | Unclear risk^a^ | Unclear risk^a^ | Unclear risk | Low risk | Low risk | Low risk | Low risk |
| Ibiloye et al. | Unclear risk^a^ | Unclear risk^a^ | Unclear risk | Low risk | Low risk | Low risk | Low risk |
| Kaplan et al. | Unclear risk^a^ | Unclear risk^a^ | Unclear risk | Low risk | Low risk | Low risk | Low risk |
| Katz et al. | Unclear risk^a^ | Unclear risk^a^ | Unclear risk | Low risk | Low risk | Low risk | Low risk |
| Kiguba et al. | Unclear risk^a^ | Unclear risk^a^ | Unclear risk | Low risk | Low risk | Low risk | Low risk |
| Mberi et al. | Unclear risk^a^ | Unclear risk^a^ | Unclear risk | Low risk | Low risk | Low risk | Low risk |
| Megerso et al. | Unclear risk^a^ | Unclear risk^a^ | Unclear risk | Low risk | Low risk | Low risk | Low risk |
| Mekonnen et al. | Unclear risk^a^ | Unclear risk^a^ | Unclear risk | Low risk | Low risk | Low risk | Low risk |
| Melaku et al. | Unclear risk^a^ | Unclear risk^a^ | Unclear risk | Low risk | Low risk | Low risk | Low risk |
| Meloni et al. | Unclear risk^a^ | Unclear risk^a^ | Unclear risk | Low risk | Low risk | Low risk | Low risk |
| Mugisha et al. | Unclear risk^a^ | Unclear risk^a^ | Unclear risk | Low risk | Low risk | Low risk | Low risk |
| Seifu et al. | Unclear risk^a^ | Unclear risk^a^ | Unclear risk | Low risk | Low risk | Low risk | Low risk |
| Tadesse et al. | Unclear risk^a^ | Unclear risk^a^ | Unclear risk | Low risk | Low risk | Low risk | Low risk |
| Teshome et al. | Unclear risk^a^ | Unclear risk^a^ | Unclear risk | Low risk | Low risk | Low risk | Low risk |
| Tweya et al. | Unclear risk^a^ | Unclear risk^a^ | Unclear risk | Low risk | Low risk | Low risk | Low risk |
| Van Cutsem et al. | Unclear risk^a^ | Unclear risk^a^ | Unclear risk | Low risk | Low risk | Low risk | Low risk |
| Wang et al. | Unclear risk^a^ | Unclear risk^a^ | Unclear risk | Low risk | Low risk | Low risk | Low risk |

^a^ = Not applicable due to type of study design
